# Self-rated attractiveness predicts preferences for sexually dimorphic facial characteristics in a culturally diverse sample

**DOI:** 10.1038/s41598-021-90473-3

**Published:** 2021-05-25

**Authors:** Urszula M. Marcinkowska, Benedict C. Jones, Anthony J. Lee

**Affiliations:** 1 Faculty of Health Sciences, Jagiellonian University Medical College, Cracow, Poland; 2 School of Psychological Sciences and Health, University of Strathclyde, Glasgow, Scotland, UK; 3 Division of Psychology, Faculty of Natural Sciences, University of Stirling, Stirling, Scotland, UK

**Keywords:** Psychology and behaviour, Sexual selection

## Abstract

Individuals who are more attractive are thought to show a greater preference for facial sexual dimorphism, potentially because individuals who perceive themselves as more physically attractive believe they will be better able to attract and/or retain sexually dimorphic partners. Evidence for this link is mixed, however, and recent research suggests the association between self-rated attractiveness and preferences for facial sexual dimorphism may not generalise to non-Western cultures. Here, we assess whether self-rated attractiveness and self-rated health predict facial sexual dimorphism preferences in a large and culturally diverse sample of 6907 women and 2851 men from 41 countries. We also investigated whether ecological factors, such as country health/development and inequality, might moderate this association. Our analyses found that men and women who rated themselves as more physically attractive reported stronger preferences for exaggerated sex-typical characteristics in other-sex faces. This finding suggests that associations between self-rated attractiveness and preferences for sexually dimorphic facial characteristics generalise to a culturally diverse sample and exist independently of country-level factors. We also found that country health/development moderated the effect of men’s self-rated attractiveness on femininity preferences, such that men from countries with high health/development showed a positive association between self-rated attractiveness and femininity preference, while men from countries with low health/development showed the opposite trend.

## Introduction

Sexually dimorphic characteristics in men’s and women’s faces are thought to influence attractiveness judgments because they signal underlying characteristics that predict men’s and women’s reproductive potential^1,2^. For example, masculine characteristics in men’s faces and feminine characteristics in women’s faces are commonly assumed to be positively correlated with heritable aspects of men’s and women’s health^1,2^, although results from empirical tests of this assumption are equivocal^3–9^. While questions have been raised about the putative link between sexually dimorphic facial characteristics and health, multiple lines of evidence have demonstrated that men and women with feminine characteristics are perceived to possess prosocial characteristics that would be beneficial to relationships and offspring, such as trustworthiness, emotional warmth, and being a good parent^10,11^. Moreover, there is some evidence that these trait attributions may partly reflect positive correlations between facial femininity and actual behavior in women (e.g.,^12^).

Several recent studies have reported that the extent to which men and women prefer other-sex faces displaying exaggerated sex-typical characteristics covaries with how physically attractive the perceiver believes themself to be (^13–16^, but see also^17,18^). This association between self-rated attractiveness and face preferences is thought to occur because individuals who believe that they are physically attractive also believe they will be able to attract, retain, and/or replace high-quality mates^13–16^. Consistent with this interpretation, experimentally manipulating women’s beliefs about their own attractiveness directly influences their preferences for masculine men^19^ and self-rated attractiveness is a better predictor of women’s masculinity preferences than are objective measures of their physical attractiveness^14^.

Evidence for positive correlations between self-rated attractiveness and both women’s preferences for masculine men and men’s preferences for feminine women has typically come from large online studies of western men’s and women’s face preferences. It is therefore an open question whether such associations occur in more culturally diverse samples. Indeed, a recent study of Arab women’s face preferences found no evidence that Arab women who considered themselves to be particularly attractive showed stronger preferences for masculine men^20^. Moreover, some researchers have suggested that correlations between self-rated attractiveness and preferences for sexually dimorphic facial characteristics could simply be a by-product of regional variation in face preferences and self-rated attractiveness^14,17^.

In addition to the association between self-rated attractiveness and face preferences, self-rated health might also predict mate preferences. Feinberg et al.^21^ reported that self-rated health was negatively associated with women’s preferences for masculine characteristics in recordings of men’s voices^21^, potentially because women who consider themselves to be particularly healthy can better offset the costs (e.g., increased risk of contracting infectious illnesses) of choosing a mate prone to infectious illnesses.

In light of the above, we investigated the relationship between self-rated attractiveness and self-rated health, and preferences for sexually dimorphic facial characteristics in a large, culturally diverse sample. We addressed the question whether associations between self-rated attractiveness/self-rated health and preferences for sexually dimorphic facial cues occur independently of regional differences in face preferences in two ways. First, we used linear mixed models to control for regional variation in face preferences that occurred at the country level. Second, we controlled for country-level factors that have been found to predict regional variation in preferences for sexually dimorphic facial characteristics in previous research, such as environmental harshness (in the form of country health/development) and country inequality^22–26^. In addition to investigating whether self-rated attractiveness predicted preferences for sexually dimorphic facial characteristics independent of these environmental factors, we also investigated whether environmental factors moderated the effects of self-rated attractiveness on preferences for sexually dimorphic facial cues. We had no specific a priori predictions for this exploratory moderation analysis.

## Methods

### Participants

Participants were recruited via various web pages (e.g., university websites and social media) and through information boards advertising the online study^27^. Due to difficulties in internet access in Nigeria and Nepal, the survey was promoted and conducted by local collaborators. Data were collected from 8957 women and 3473 men from a total of 116 countries. Analyzed data were taken from the dataset gathered to investigate predictors of regional and individual variation in face preferences^25,27^.

Participants were excluded from the dataset prior to analyses if they did not report being exclusively heterosexual (N = 3397), or, following Lee, DeBruine and Jones^28^, if the total number of participants in a country was less than 10 (N = 158). These exclusions resulted in a final sample of 6907 women and 2851 men (mean age = 27.92 years, SD = 9.37 years) from 41 countries. The countries included in the analyzed sample were Argentina, Australia, Belgium, Brazil, Canada, China, Colombia, Croatia, Czech Republic, Denmark, Estonia, Finland, France, Germany, Iceland, Iran, Ireland, Italy, Japan, Latvia, Malaysia, Mexico, Nepal, Netherlands, New Zealand, Nigeria, Norway, Poland, Portugal, Romania, Russian Federation, Saudi Arabia, Singapore, Slovakia, Spain, Sweden, Switzerland, Turkey, Ukraine, United Kingdom, and United States of America.

### Survey and stimuli

Participants completed an online survey that consisted of two blocks. The first block consisted of a questionnaire assessing socio-demographic information and self-rated attractiveness and self-rated health. Participants answered the questions “How attractive would you say you are?” and “How healthy would you say you are?” on a scale from − 3 “Very unattractive/unhealthy (far below average)” to + 3 “Very attractive/healthy (far above average)”.

The second block consisted of a two alternative forced choice task, where participants were presented with pairs of facial photographs that were manipulated along the femininity-masculinity dimension. Images of male and female adult White faces (between 18 and 24 years old) were manipulated using established computer graphics methods^29^. Face images were manipulated along the femininity–masculinity continuum by adding or subtracting 50% of the linear differences in shape between a male facial composite (i.e., average male face) and a female facial composite (i.e., average female face), following procedures from previous research^23,27^.

Following previous research on variation in face preferences [e.g.,^13–15,18^], participants were presented with two versions of twenty other-sex faces (i.e., the masculinized and feminized versions) and were asked to choose the face in each pair that they perceived to be the more attractive. Trial order and onscreen face position (left vs. right hand side) were both fully randomized. Forced-choice paradigms can produce qualitatively different patterns of results to other methods for assessing perceptions of faces^30^. However, we used the forced choice method in the current study to allow our results to be directly compared with previous research investigating putative links between self-rated attractiveness and face preferences. The survey was translated into 14 different languages and participants completed the survey in their preferred language.

### Country-level variables

To assess whether country-level factors moderate the association between preference for masculinity and self-rated attractiveness and self-rated health, we computed a country health/development factor and an inequality factor based on country statistics via an Independent Factor Analysis (IFA) as reported in Marcinkowska et al.^25^ and Marcinkowska et al.^31^. The IFA originally included 121 countries to develop the factor scores; from this, the factor scores for the 41 countries included in this sample were taken. The IFA included 11 country demographic statistics, including the Human Development Index, life expectancy, years lost to disease, fertility rate, the Gender Inequality Index (GII), urbanisation, historical pathogen prevalence, mortality rate, homicide rate, Gini coefficient, and GDP. The IFA resulted in two factors, a health/development factor, and an inequality factor, which were included as predictors in in our analyses. Higher scores on the health/development factor indicated greater health/development, while higher scores on the inequality factor indicated greater inequality.

### Statistical analysis

The data were analysed using binomial mixed effects modelling in the R statistical program^32^ using the lme4^33^ and lmerTest^34^ packages. We conducted a model with age, self-rated attractiveness, and self-rated health as fixed effects, with responses on each trial as the outcome variable (0 = less sexually dimorphic face chosen, 1 = more sexually dimorphic face chosen). To test whether the association between self-rated attractiveness and self-rated health are moderated by country-level factors, we also included the health/development factor and inequality factor from the IFA, and their interactions with age, self-rated attractiveness, and self-rated health as fixed effects. All predictors were *z*-standardised at the appropriate group-level (i.e., participant variables such as age, self-rated attractiveness, and self-rated health are standardised across all participant, while country-level variables, such as the health/development and inequality factors, are standardised by country). Outliers were winsorised to ± 3SDs from the mean. To account for non-independence, random intercepts were specified for each participant, stimulus, country and region. Random slopes were specified maximally following the recommendations in Barr, Levy, Scheepers and Tily^35^ and Barr^36^. Data for male and female participants were analysed in separate models. The estimated fixed effects are reported here; for full model specifications and output (including estimated random effects), see the Supplementary Materials S1. The dataset and analysis code supporting this article can be accessed at osf.io/6aeyq.

Correlations between individual-level predictors for both men and women were less than 0.46, indicating that multicollinearity was not an issue (see the Supplementary Materials S1 for full details).

### Ethical approval

The project was approved by the Ethics in Research Committee of Daugavpils University, Lativia in accordance to their guidelines and regulations. All participants provided informed consent before participating in the study.

## Results

### Women’s preference for masculinity in male faces

**Figure 1:**
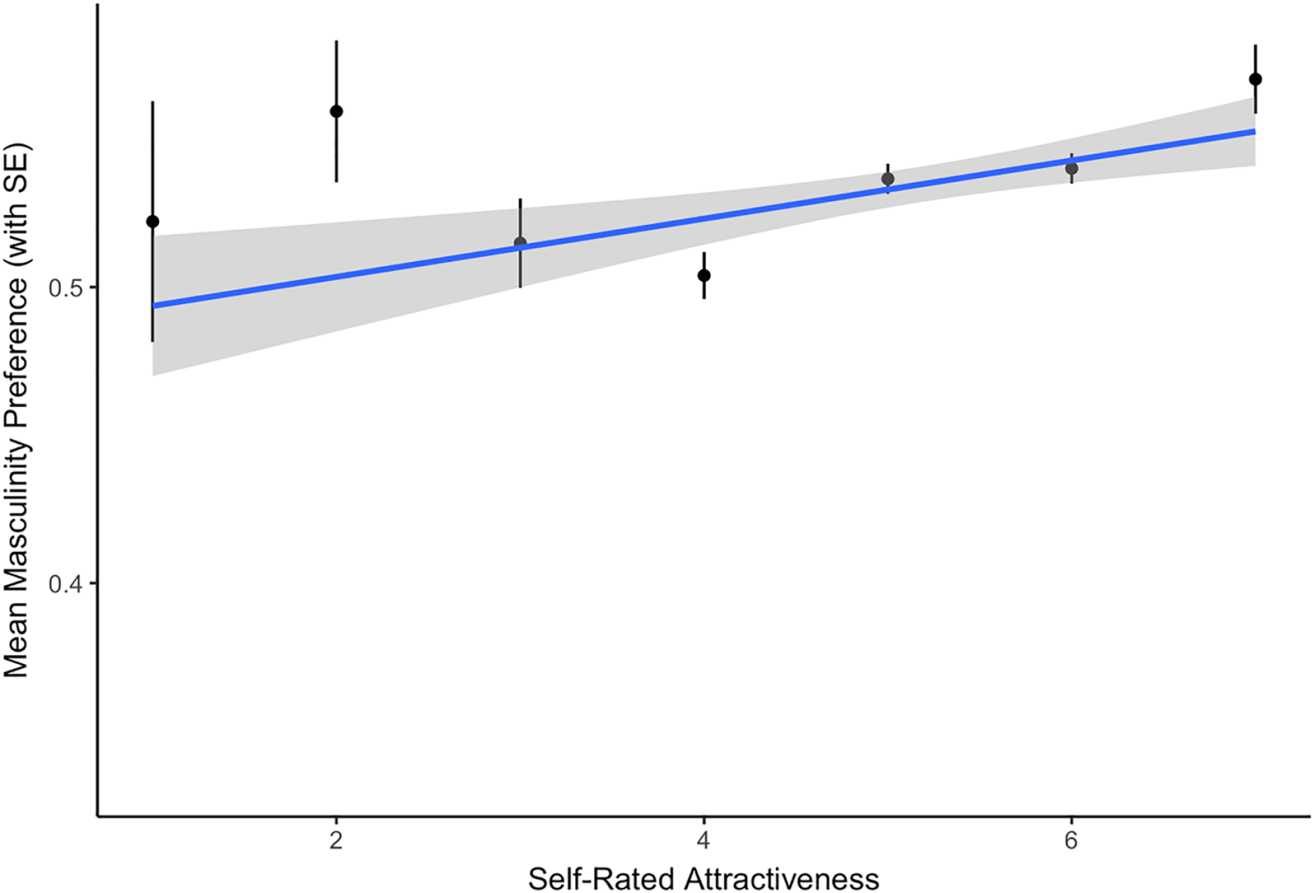
The significant association between self-rated attractiveness and women’s preferences for male facial masculinity.

**Figure 2:**
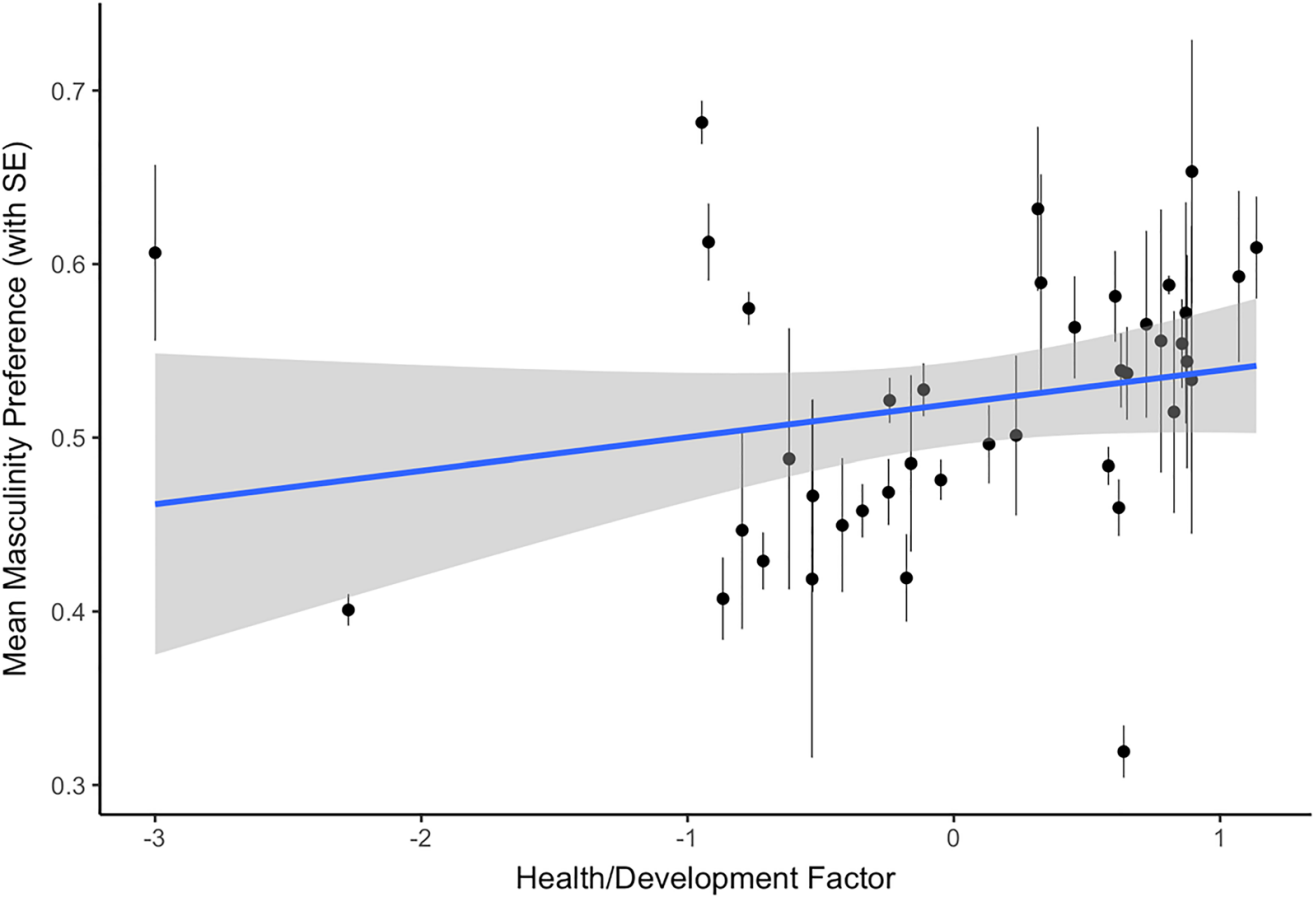
The significant association between scores on the health/development factor and women’s preferences for male facial masculinity.

**Figure 3:**
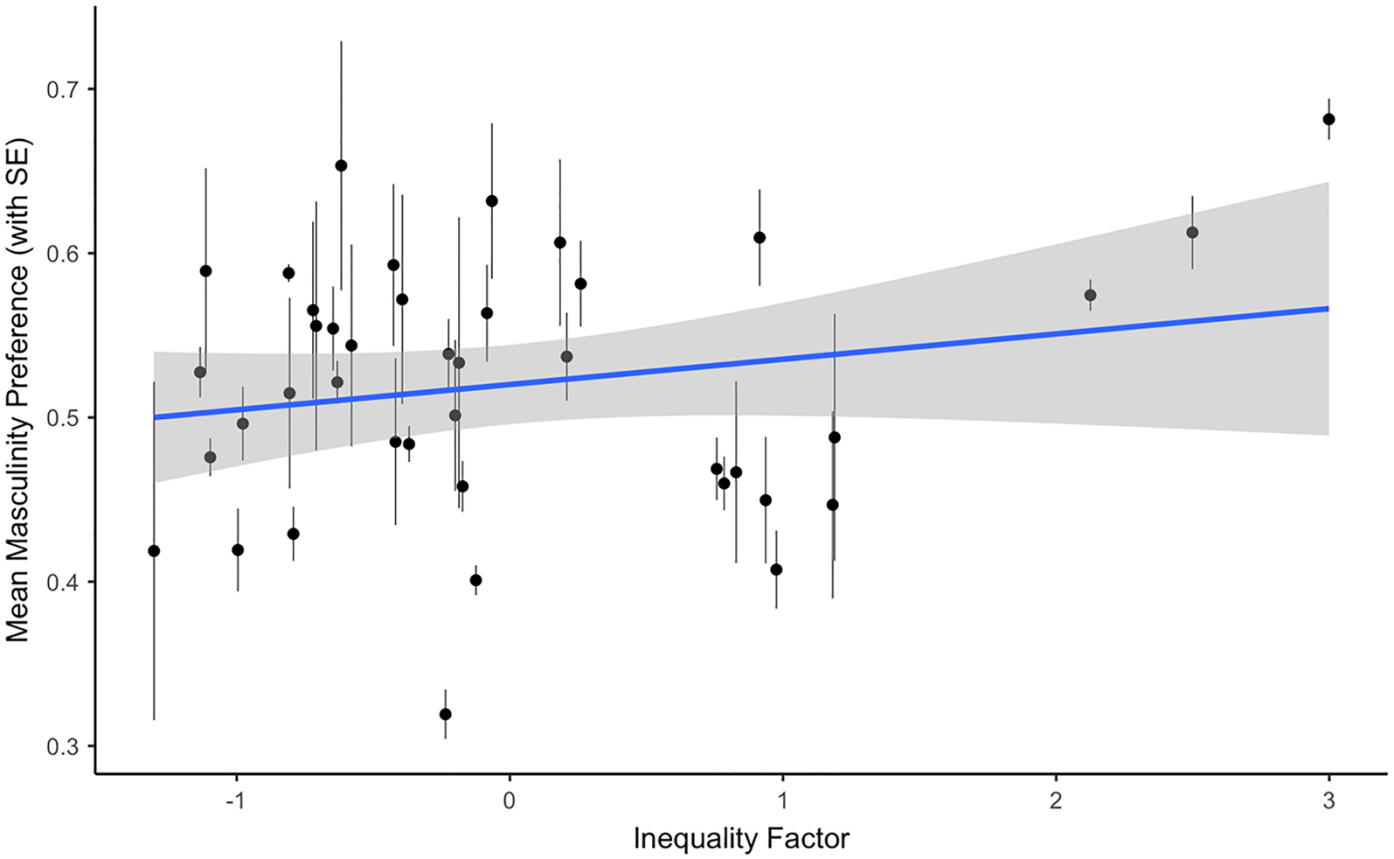
The significant association between scores on the inequality factor and women’s preferences for male facial masculinity.

The estimated fixed effects for the model of women’s preference for masculinity in male faces are reported in Table 1. Consistent with previous findings, there was a positive association between women’s self-rated attractiveness and their preference for facial masculinity (see Fig. 1). There was no significant main effect of self-rated health. There was also a positive association between the health/development factor and preference for masculinity (see Fig. 2), as well as between the inequality factor and preference for masculinity (see Fig. 3). Country-level factors did not significantly interact with self-rated attractiveness and there were no significant effects involving self-rated health.

**Table 1:** The estimated fixed effects for the model of women’s preference for masculinity in male faces.

	Estimate (std. error)	*z*-value	*p*-value
**Intercept**	0.08 (0.31)	0.24	0.808
Age	0.16 (0.04)	4.03	< 0.001
Self-rated attractiveness	0.08 (0.02)	3.64	< 0.001
Self-rated health	0.00 (0.02)	0.02	0.985
**Country health/development**	0.23 (0.10)	2.34	0.019
x Age	− 0.07 (0.07)	− 0.89	0.375
x Self-rated attractiveness	0.03 (0.03)	0.79	0.427
x Self-rated health	− 0.01 (0.03)	− 0.40	0.693
**Country inequality**	0.19 (0.10)	2.05	0.040
x Age	− 0.00 (0.04)	− 0.03	0.973
x Self-rated attractiveness	0.01 (0.02)	0.40	0.688
x Self-rated health	− 0.01 (0.02)	− 0.56	0.573

### Men’s preference for femininity in female faces

**Figure 4:**
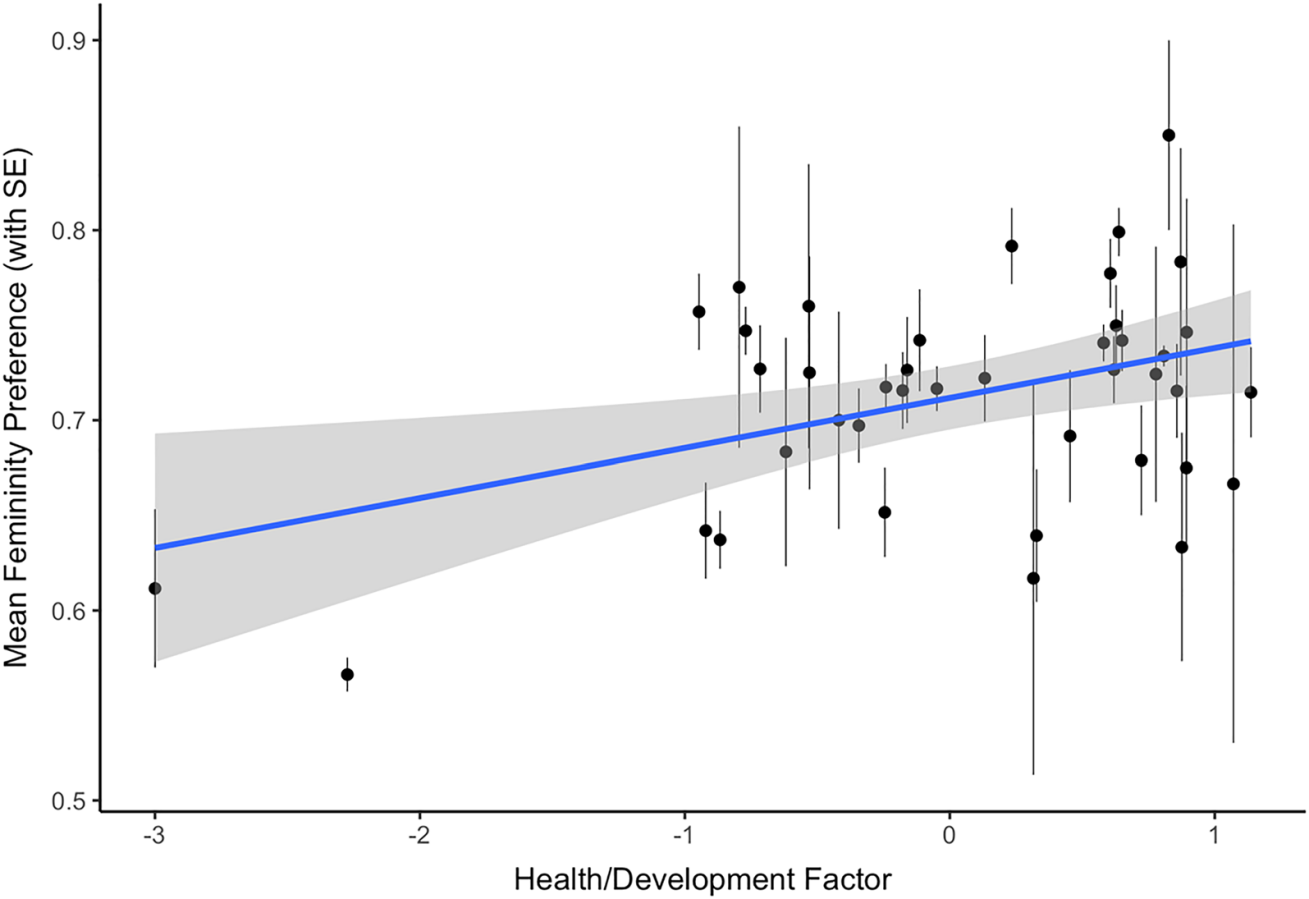
The significant association between scores on the health/development factor and men’s preferences for female facial femininity.

**Figure 5:**
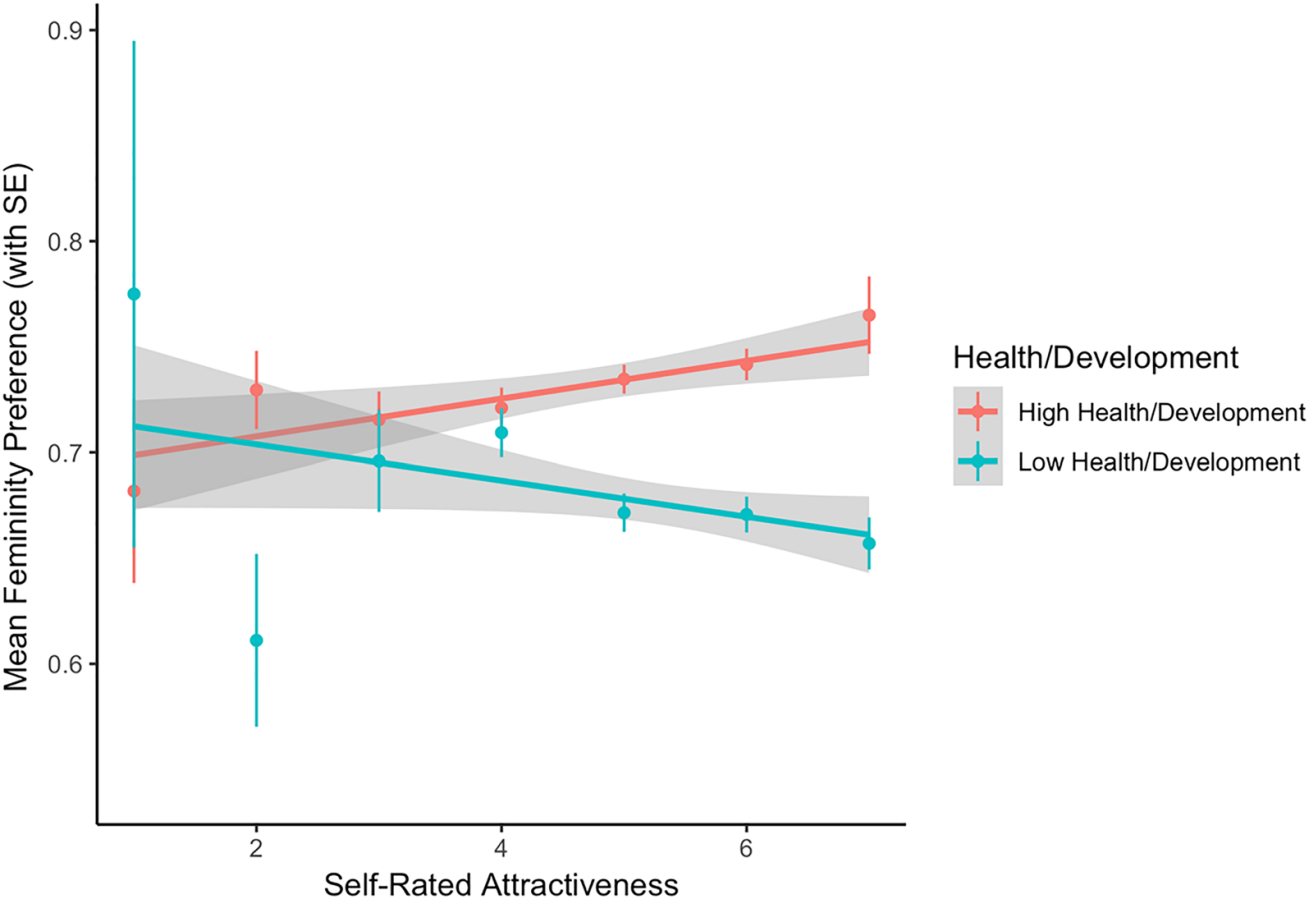
The significant interaction between the effects of scores on the health/development factor and men’s self-rated attractiveness.

The estimated fixed effects for the model of men’s preference for femininity in female faces are reported in Table 2. There was a significant, positive association between self-rated attractiveness and facial femininity preference, such that, as self-rated attractiveness increased, men showed a greater preference for facial femininity. There was also a significant main effect of country health/development (see Fig. 4). Country health/development also significantly moderated the association between self-rated attractiveness and femininity preference; men from countries with high health/development showed a positive association between self-rated attractiveness and femininity preference, while men from countries with low health/development, self-rated attractiveness was negatively associated with preference for facial femininity (see Fig. 5). There were no significant effects involving self-rated health or the country inequality factor.

**Table 2:** The estimated fixed effects for the model of men’s preference for facial femininity in female faces.

	Estimate (std. error)	*z*-value	*p*-value
**Intercept**	1.08 (0.25)	4.35	< 0.001
Age	− 0.11 (0.02)	− 4.71	< 0.001
Self-rated attractiveness	0.06 (0.02)	2.97	0.003
Self-rated health	− 0.00 (0.02)	− 0.08	0.936
**Country health/development**	0.15 (0.05)	2.74	0.006
x Age	− 0.01 (0.03)	− 0.47	0.640
x Self-rated attractiveness	0.05 (0.02)	2.43	0.015
x Self-rated health	− 0.03 (0.02)	− 1.12	0.262
**Country inequality**	0.03 (0.07)	0.39	0.695
x Age	− 0.02 (0.02)	− 0.91	0.365
x Self-rated attractiveness	0.03 (0.02)	1.27	0.205
x Self-rated health	0.02 (0.02)	0.99	0.322

## Discussion

Our analyses showed that men and women who rated themselves as more physically attractive reported stronger preferences for exaggerated sex-typical characteristics in other-sex faces. In other words, self-rated attractiveness was positively correlated with men’s preferences for femininity in women’s faces and women’s preferences for masculinity in men’s faces. These correlations are consistent with previous studies reporting positive correlations between self-rated attractiveness and preferences for sexually dimorphic facial characteristics [^13–16^, but see also^17,18^]. By contrast with Feinberg et al.^21^ work on women’s preferences for male vocal masculinity, our analyses of face preferences found no evidence that women who rated their health to be particularly good showed weaker preferences for masculine faces.

Importantly, and in contrast to previous research, here we show associations between self-rated attractiveness and preferences for sexually dimorphic facial characteristics in a culturally diverse sample. Furthermore, our analyses showed that the effect of self-rated attractiveness was independent of country-level variation in environmental harshness and inequality. These results do not support the proposal that the effects of self-rated attractiveness on preferences for sexually dimorphic facial characteristics reported in previous work may simply be a by-product of country-level differences in face preferences and self-rated attractiveness^14,17^.

In addition to the effects of self-rated attractiveness described above, we also found that men and women in countries with harsher environments (as indicated by scores on a health/development factor) showed weaker preferences for feminine women and masculine men, respectively. This could suggest that, under certain ecological conditions, the importance of exaggerated sexual dimorphism in a putative partner is diminished. For instance, in harsher environments, women may place a greater importance on a partner with good parental ability, typically associated with less masculine male faces^37^, while men may favour more masculine women as physical dominance (associated with increased masculinity) may be more beneficial^38^). These results are consistent with those from some previous studies of regional variation in face preferences^26^, but do not support the proposal that people show stronger preferences for other-sex faces displaying exaggerated sex-typical characteristics in harsher environments^23,24^. We also found that women showed stronger preferences for masculine men in countries with high inequality, replicating previous research^22^.

Interestingly, scores on the health/development factor also moderated the effect of men’s self-rated attractiveness on femininity preferences. Self-rated attractiveness was positively correlated with femininity preferences in countries that scored high on the health/development factor, but not in countries that scored low on the health/development factor. This result was not an a priori prediction, but suggests that the effect of self-rated attractiveness on men’s face preferences is potentially not consistent across environments. It is possible that men in countries with poorer health simply do not calibrate their preferences to their perceptions of their market value under such conditions, instead simply looking to maximise the quantity (rather than quality) of mates they obtain.

A possible limitation of the gathered data was that all participants were presented with visual stimuli of White faces. This was an effect of a trade-off between maximising the cultural variation within the tested sample and adjusting surveys to local environments. Previous work has also suggested that distinctiveness can influence facial attractiveness; as such, we could expect the extent to which facial sexual dimorphism exists in a given culture may bias responses. Future studies could investigate whether the ethnicity of presented faces changes the observed moderation effect.

In summary, our results add to a growing body of evidence implicating self-rated attractiveness and ecological factors in men’s and women’s preferences for sexually dimorphic facial characteristics. Our results also suggest that the association between self-rated attractiveness and facial preferences are not simply a by-product of two independent sets of factors (regional variation in self-rated attractiveness and face preferences), as some researchers have suggested. Our work also examines the interaction between ‘market demands’ (in the form of an individual’s attractiveness) and biological markets, and suggests both individual characteristics and ecology can moderate mate preferences. Future work could investigate whether this helps explain the mixed evidence for a correlation between mate preference and mate choice.

## Supplementary Information

Supplementary Information.

## References

[1] Little AC, Jones BC, DeBruine LM (2011). Facial attractiveness: Evolutionary based research. Philos. Trans. R. Soc. B Biol. Sci..

[2] Thornhill R, Gangestad SW (1999). Facial attractivness. Trends Cogn. Sci..

[3] Boothroyd LG (2013). Male facial masculinity as a cue to health outcomes. Evol. Psychol..

[4] Foo, Y. Z., Simmons, L. W., & Rhodes, G. The relationship between health and mating success in humans. *R. Soc. Open Sci.* **4**, 160603 (2017). 10.1098/rsos.160603 PMC5319324 28280558

[5] Cai, Z., *et al.* No evidence that facial attractiveness, femininity, averageness, or coloration are cues to susceptibility to infectious illnesses in a university sample of young adult women. *Evol. Hum. Behav. ***40**(2), 156–159 (2019).

[6] Gray AW, Boothroyd LG (2012). Female facial appearance and health. Evol. Psychol..

[7] Thornhill R, Gangestad SW (2006). Facial sexual dimorphism, developmental stability, and susceptibility to disease in men and women. Evol. Hum. Behav..

[8] Rhodes G (2003). Does sexual dimorphism in human faces signal health?. Proc. R. Soc. Lond. B.

[9] Zaidi, A. A., *et al.* Facial masculinity does not appear to be a condition-dependent male ornament and does not reflect MHC heterozygosity in humans. *Proc. Natl. Acad. Sci. ***116**(5), 1633–1638 (2019). 10.1073/pnas.1808659116 PMC6358690 30647112

[10] Boothroyd LG (2007). Partner characteristics associated with masculinity, health and maturity in male faces. Pers. Individ. Differ..

[11] Perrett DI (1998). Effects of sexual dimorphism on facial attractiveness. Nature.

[12] Law Smith MJ (2012). Maternal tendencies in women are associated with estrogen levels and facial femininity. Horm. Behav..

[13] Batres, C. M., Kannan, M., & Perrett, D. I. Familiarity with own population's apperance influences facial preferences. *Hum. Nat. ***28**, 344–354 (2017). 10.1007/s12110-017-9289-8 PMC5524856 28516361

[14] Docherty C (2020). Do more attractive women show stronger preferences for male facial masculinity?. Evol. Hum. Behav..

[15] Kandrik M, DeBruine LM (2012). Self-rated attractiveness predicts preferences for opposite-sex faces, while self-rated sex-typicality predicts preferences for same-sex faces. J. Evol. Psychol..

[16] Little AC (2001). Self-perceived attractiveness influences human female preferences for sexual dimorphism and symmetry in male faces. Proc. R. Soc. Lond. Ser. B Biol. Sci..

[17] Penton-Voak IS (2003). Female condition influences preferences for sexual dimorphism in faces of male humans (Homo sapiens). J. Comp. Psychol..

[18] Zietsch BP (2015). Variation in women's facial masculinity preference is better explained by genetic differences than by previously identified context-dependent effects. Psychol. Sci..

[19] Little AC, Mannion H (2006). Viewing attractive or unattractive same-sex individuals changes self-rated attractiveness and face preferences in women. Anim. Behav..

[20] Alharbi, S. A. H., *et al.* Does self-rated attractiveness predict women's preferences for facial masculinity? Data from an Arab sample. *Adapt. Hum. Behav. Physiol. ***7**, 105–113 (2021).

[21] Feinberg DR (2012). Women’s self-perceived health and attractiveness predict their male vocal masculinity preferences in different directions across short- and long-term relationship contexts. Behav. Ecol. Sociobiol..

[22] Brooks R (2011). National income inequality predicts women's preferences for masculinized faces better than health does. Proc. R. Soc. B Biol. Sci..

[23] DeBruine LM (2010). The health of a nation predicts their mate preferences: Cross-cultural variation in women's preferences for masculinized male faces. Proc. R. Soc. B Biol. Sci..

[24] DeBruine LM (2010). Further evidence for regional variation in women's masculinity preferences. Proc. R. Soc. B Biol. Sci..

[25] Marcinkowska UM (2019). Women's preferences for men's facial masculinity are strongest under favorable ecological conditions. Sci. Rep..

[26] Scott, I. M. L., *et al.* Does masculinity matter? The contribution of masculine face shape to male attractiveness in humans. *PLoS ONE* **5**(10), e13585 (2010). 10.1371/journal.pone.0013585 PMC2965103 21048972

[27] Marcinkowska UM (2014). Cross-cultural variation in men's preference for sexual dimorphism in women's faces. Biol. Lett..

[28] Lee AJ, DeBruine LM, Jones BC (2018). Individual-specific mortality is associated with how individuals evaluate future discounting decisions. Proc. R. Soc. B Biol. Sci..

[29] Tiddeman B, Burt M, Perrett D (2001). Prototyping and transforming facial textures for perception research. IEEE Comput. Graphics Appl..

[30] Jones AL, Jaeger B (2019). Biological bases of beauty revisited: The effect of symmetry, averageness, and sexual dimorphism on female facial attractiveness. Symmetry.

[31] Marcinkowska, U.M., et al., Exploratory study of variability in perception of putative fertility facial cues – Cross-cultural and methodological approach. (In Prep).

[32] R Core Team, R: *A language and environmental for statistical computing.* 2013, Vienna, Austria: R Foundation for Statistical Computing.

[33] Bates D (2015). Fitting linear mixed-effects models usng lme4. J. Stat. Softw..

[34] Kuznetsova, A., P.B. Brockhoff, and R.H.B. Christensen. *lmerTest: Tests for random and fixed effects for linear mexed effect models.* 2015; Available from: https://CRAN.R-project.org/package=lmerTest.

[35] Barr DJ (2013). Random effects structure for confirmatory hypothesis testing: Keep it maximal. J. Mem. Lang..

[36] Barr DJ (2013). Random effects structure for testing interactions in linear mixed-effects models. Front. Psychol..

[37] Kruger DJ (2006). Male facial masculinity influences attributions of personality and reproductive strategy. Pers. Relat..

[38] Penton-Voak IS, Jacobson A, Trivers R (2004). Populational differences in attractiveness judgements of male and female faces: Comparing British and Jamaican samples. Evol. Hum. Behav..

