# Supplementary material for: Self-rated attractiveness predicts preferences for sexually dimorphic facial characteristics in a culturally diverse sample
Source: Sci Rep. 2021 May 25;11:10905. doi: 10.1038/s41598-021-90473-3 (PMC8149859; doi:10.1038/s41598-021-90473-3)
Supplement: Supplementary file 1 — Supplementary Information. [file 41598_2021_90473_MOESM1_ESM.pdf]

Self-rated attractiveness predicts preferences for sexually dimorphic facial characteristics: Evidence from a culturally diverse sample

Contents

Note: Code for data preparation is included below, though not necessary to replicate the data. . . 1

Load Packages and Custom Functions **1**

Load Data **2**
Recode Country Values . . . 2
Load Data . . . 2

Participant Demographics **2**

Prepare Data **2**
Participant Data . . . 2
Rating Data . . . 6
Country Data . . . 6

Analyses - Is Self-Rated Attractiveness and Self-Rated Health Associated with Masculinity Preferences **7**
Female Participants rating Male Faces . . . 7
Male Participants rating Female Faces . . . 9

Analysis with Ecological Factors **12**
Female Participants rating Male Faces . . . 12
Male Analysis . . . 18

Note: Code for data preparation is included below, though not necessary to replicate the data.

Load Packages and Custom Functions

```
library(tidyverse)
library(lme4)
library(lmerTest)

checkN <- function(data,label){
  print(paste(label,": ",NROW(data),sep = ""))
  return(data)
}

z <- function(x,remove.outliers = FALSE,winsorise = FALSE){
    out <- (x - mean(x,na.rm = TRUE))/sd(x,na.rm = TRUE)
    if (remove.outliers == TRUE){
      out <- ifelse(out >3,NA,ifelse(out < -3,NA,out))
```

```
    }
    if (winsorise == TRUE){
      out <- ifelse(out > 3,3,ifelse(out < -3,-3,out))
    }
    return(out)
}
```

Load Data

Recode Country Values

```
country.codes <- read.csv("Country List.csv",stringsAsFactors = FALSE) %>%
    mutate( code = as.numeric(gsub("[^[:digit:]]","",country)),
            country = gsub("[[:digit:]]","",country),
            country = trimws(country))
```

Load Data

```
full.data <- read.csv("full.data.csv",stringsAsFactors = FALSE)
```

Participant Demographics

```
#Number of men and women in full sample
table(full.data$sex)
```

```
##
## Female   Male
##   8957   3473
```

```
#Total number of countries in full sample
NROW(unique(full.data$country))
```

```
## [1] 116
```

```
#Mean and SD for age in full sample
summarise(full.data, mean.age = mean(age,na.rm = TRUE),
                sd.age = sd(age,na.rm = TRUE))
```

```
##   mean.age   sd.age
## 1   27.624 9.172805
```

Prepare Data

Participant Data

```r
participant.data <- full.data %>%
  select(ResponseId,country,sex,age,sexo,rs,sra,srh) %>% checkN("Full Sample") %>%
    filter(sexo == 1) %>% checkN("Exclusively Heterosexual") %>%
  filter(!is.na(country)) %>%
    group_by(country) %>%
    filter(n() >= 10) %>% checkN("More than 10 participants per country") %>%
  ungroup() %>%
    mutate( z.age = z(age,winsorise = TRUE),
            z.sra = z(sra,winsorise = TRUE),
            z.srh = z(srh,winsorise = TRUE))
```

```
## [1] "Full Sample: 13313"
## [1] "Exclusively Heterosexual: 9916"
## [1] "More than 10 participants per country: 9758"
```

```r
#Number of men and women in full sample
table(participant.data$sex)
```

```
##
## Female   Male
##   6907   2851
```

```r
#Total number of countries in full sample
NROW(unique(participant.data$country))
```

```
## [1] 41
```

```r
sort(unique(participant.data$country))
```

```
##  [1] "Argentina"
##  [2] "Australia"
##  [3] "Belgium"
##  [4] "Brazil"
##  [5] "Canada"
##  [6] "China"
##  [7] "Colombia"
##  [8] "Croatia"
##  [9] "Czech Republic"
## [10] "Denmark"
## [11] "Estonia"
## [12] "Finland"
## [13] "France"
## [14] "Germany"
## [15] "Iceland"
## [16] "Iran, Islamic Republic of..."
## [17] "Ireland"
## [18] "Italy"
## [19] "Japan"
## [20] "Latvia"
## [21] "Malaysia"
## [22] "Mexico"
## [23] "Nepal"
## [24] "Netherlands"
## [25] "New Zealand"
## [26] "Nigeria"
## [27] "Norway"
```

```
## [28] "Poland"
## [29] "Portugal"
## [30] "Romania"
## [31] "Russian Federation"
## [32] "Saudi Arabia"
## [33] "Singapore"
## [34] "Slovakia"
## [35] "Spain"
## [36] "Sweden"
## [37] "Switzerland"
## [38] "Turkey"
## [39] "Ukraine"
## [40] "United Kingdom of Great Britain and Northern Ireland"
## [41] "United States of America"
```

```
#Mean and SD for age in full sample
summarise(participant.data, mean.age = mean(age,na.rm = TRUE),
                sd.age = sd(age,na.rm = TRUE))
```

```
## # A tibble: 1 x 2
##   mean.age sd.age
##      <dbl>  <dbl>
## 1     27.9   9.37
```

Separate by participant sex and check for multicollinearity

Male Participants

```
male.data <- participant.data %>%
    filter(sex == "Male") %>%
    mutate( z.age = z(age,winsorise = TRUE),
            z.sra = z(sra,winsorise = TRUE),
            z.srh = z(srh,winsorise = TRUE))

cor.test(male.data$age,male.data$sra)
```

```
##
##  Pearson's product-moment correlation
##
## data:  male.data$age and male.data$sra
## t = -1.8744, df = 2647, p-value = 0.06098
## alternative hypothesis: true correlation is not equal to 0
## 95 percent confidence interval:
##  -0.074389204  0.001678067
## sample estimates:
##         cor
## -0.03640831
```

```
cor.test(male.data$sra,male.data$srh)
```

```
##
##  Pearson's product-moment correlation
##
## data:  male.data$sra and male.data$srh
## t = 27.001, df = 2643, p-value < 2.2e-16
## alternative hypothesis: true correlation is not equal to 0
```

```
## 95 percent confidence interval:
##  0.4345669 0.4943310
## sample estimates:
##       cor
## 0.4649785
```

```
cor.test(male.data$age,male.data$srh)
```

```
##
##  Pearson's product-moment correlation
##
## data:  male.data$age and male.data$srh
## t = -1.2679, df = 2645, p-value = 0.2049
## alternative hypothesis: true correlation is not equal to 0
## 95 percent confidence interval:
##  -0.06268615  0.01346449
## sample estimates:
##         cor
## -0.02464658
```

Female Participants

```
female.data <- participant.data %>%
    filter(sex == "Female") %>%
    mutate( z.age = z(age,winsorise = TRUE),
            z.sra = z(sra,winsorise = TRUE),
            z.srh = z(srh,winsorise = TRUE))

cor.test(female.data$age,female.data$sra)
```

```
##
##  Pearson's product-moment correlation
##
## data:  female.data$age and female.data$sra
## t = 0.46536, df = 6578, p-value = 0.6417
## alternative hypothesis: true correlation is not equal to 0
## 95 percent confidence interval:
##  -0.01842782  0.02989646
## sample estimates:
##         cor
## 0.005737669
```

```
cor.test(female.data$sra,female.data$srh)
```

```
##
##  Pearson's product-moment correlation
##
## data:  female.data$sra and female.data$srh
## t = 39.616, df = 6565, p-value < 2.2e-16
## alternative hypothesis: true correlation is not equal to 0
## 95 percent confidence interval:
##  0.4195181 0.4585631
## sample estimates:
##      cor
## 0.439248
```

```r
cor.test(female.data$age,female.data$srh)
```

```
##
##  Pearson's product-moment correlation
##
## data:  female.data$age and female.data$srh
## t = 3.0976, df = 6587, p-value = 0.001959
## alternative hypothesis: true correlation is not equal to 0
## 95 percent confidence interval:
##  0.01400466 0.06222730
## sample estimates:
##        cor
## 0.03813818
```

Rating Data

```r
rating.data <- select(full.data,ResponseId,m1:f20) %>%
    filter(ResponseId %in% participant.data$ResponseId) %>%
    gather(key = "faceId",value = "response",m1:f20)
```

Country Data

```r
library(psych)
country.data <- read.csv("Updated Country.Data.csv",stringsAsFactors = FALSE) %>%
  mutate(   mortality = ifelse(is.na(mortality) & na <= 2,mean(mortality,na.rm = TRUE),mortality),
            fertility = ifelse(is.na(fertility) & na <= 2,mean(fertility,na.rm = TRUE),fertility),
            yltd = ifelse(is.na(yltd) & na <= 2,mean(yltd,na.rm = TRUE),yltd),
            pathogen7 = ifelse(is.na(pathogen7) & na <= 2,mean(pathogen7,na.rm = TRUE),pathogen7),
            homicide = ifelse(is.na(homicide) & na <= 2,mean(homicide,na.rm = TRUE),homicide),
            urban = ifelse(is.na(urban) & na <= 2,mean(urban,na.rm = TRUE),urban),
            gdp = ifelse(is.na(gdp) & na <= 2,mean(gdp,na.rm = TRUE),gdp),
            hdi = ifelse(is.na(hdi) & na <= 2,mean(hdi,na.rm = TRUE),hdi),
            gini = ifelse(is.na(gini) & na <= 2,mean(gini,na.rm = TRUE),gini),
            gii = ifelse(is.na(gii) & na <= 2,mean(gii,na.rm = TRUE),gii),
            life.exp = ifelse(is.na(life.exp) & na <= 2,mean(life.exp,na.rm = TRUE),life.exp)
  ) %>%
  select(-health.fa,-inequality.fa)

d <- country.data %>%
  dplyr::select(fertility,yltd,pathogen7,homicide,urban,gdp,hdi,gini,gii,mortality,life.exp)

country.fa <- principal(d,nfactors = 2,rotate = "oblimin")

country.fa.loadings <- country.fa$loadings[1:11,1:2] %>%
    as.data.frame(row.names = row.names(.)) %>%
    rownames_to_column(var = "statistic") %>%
    arrange(desc(abs(TC1)))

country.fa.loadings
```

```
##    statistic          TC1          TC2
```

```
## 1        hdi -0.96577214 -0.008968194
## 2   life.exp -0.95937182  0.042021445
## 3       yltd  0.93450741  0.037799043
## 4  fertility  0.91425169 -0.053078297
## 5        gii  0.82615810  0.225659828
## 6      urban -0.78054822  0.249851878
## 7  pathogen7  0.63085232  0.247354059
## 8        gdp -0.27200740  0.075180591
## 9       gini  0.25925243  0.676239634
## 10 mortality  0.13127476 -0.590578993
## 11  homicide -0.05234705  0.714529156
```

```
country.fa.scores <- cbind(country.data$Country,country.data$region,country.fa$scores) %>%
    as.data.frame(stringsAsFactors = FALSE) %>%
    rename(Country = V1,region = V2,health.fa = TC1, inequality.fa = TC2) %>%
    mutate(health.fa = c(scale(as.numeric(health.fa) * -1)),
           inequality.fa = c(scale(as.numeric(inequality.fa)))) %>%
  filter(Country %in% participant.data$country) %>%
  mutate(health.fa = z(health.fa,winsorise = TRUE),
         inequality.fa = z(inequality.fa,winsorise = TRUE)) %>%
  select(Country,region,health.fa,inequality.fa)

analysis.male <- left_join(male.data,rating.data,by = "ResponseId") %>%
    left_join(country.fa.scores,by = c("country" = "Country")) %>%
  mutate(response = recode(response,"0" = 1,"1" = 0))

analysis.female <- left_join(female.data,rating.data,by = "ResponseId") %>%
    left_join(country.fa.scores,by = c("country" = "Country"))
```

Analyses - Is Self-Rated Attractiveness and Self-Rated Health Associated with Masculinity Preferences

Female Participants rating Male Faces

Linear Mixed Effects Model

```
model.f <- glmer(response ~ z.age + z.sra + z.srh +
                   (1 | ResponseId) +
                   (1 + z.age + z.sra + z.srh || faceId) +
                   (1 + z.age + z.sra + z.srh || country) +
                   (1 + z.age + z.sra + z.srh || region),data = analysis.female,family = "binomial")

save(model.f,file = "model.f.Rdata")
```

```
load("model.f.Rdata")
summary(model.f)
```

```
## Generalized linear mixed model fit by maximum likelihood (Laplace
##   Approximation) [glmerMod]
##  Family: binomial  ( logit )
## Formula:
## response ~ z.age + z.sra + z.srh + (1 | ResponseId) + (1 + z.age +
```

```
##     z.sra + z.srh || faceId) + (1 + z.age + z.sra + z.srh ||
##     country) + (1 + z.age + z.sra + z.srh || region)
##    Data: analysis.female
##
##      AIC      BIC   logLik deviance df.resid
## 131399.1 131564.0 -65682.6 131365.1   120479
##
## Scaled residuals:
##     Min      1Q  Median      3Q     Max
## -8.1261 -0.6458  0.2237  0.6280  8.4500
##
## Random effects:
##  Groups     Name        Variance  Std.Dev.
##  ResponseId (Intercept) 1.584e+00 1.259e+00
##  country    z.srh       1.295e-12 1.138e-06
##  country.1  z.sra       6.185e-04 2.487e-02
##  country.2  z.age       1.877e-02 1.370e-01
##  country.3  (Intercept) 6.946e-02 2.636e-01
##  faceId     z.srh       0.000e+00 0.000e+00
##  faceId.1   z.sra       0.000e+00 0.000e+00
##  faceId.2   z.age       7.878e-03 8.876e-02
##  faceId.3   (Intercept) 1.164e+00 1.079e+00
##  region     z.srh       0.000e+00 0.000e+00
##  region.1   z.sra       0.000e+00 0.000e+00
##  region.2   z.age       0.000e+00 0.000e+00
##  region.3   (Intercept) 1.562e-01 3.952e-01
## Number of obs: 120496, groups:
## ResponseId, 6325; country, 41; faceId, 21; region, 7
##
## Fixed effects:
##              Estimate Std. Error z value Pr(>|z|)
## (Intercept)  0.042573   0.295008   0.144 0.885255
## z.age        0.166733   0.048133   3.464 0.000532 ***
## z.sra        0.072037   0.023548   3.059 0.002220 **
## z.srh       -0.001544   0.020101  -0.077 0.938771
## ---
## Signif. codes:  0 '***' 0.001 '**' 0.01 '*' 0.05 '.' 0.1 ' ' 1
##
## Correlation of Fixed Effects:
##       (Intr) z.age  z.sra
## z.age  0.022
## z.sra -0.009  0.001
## z.srh -0.003 -0.035 -0.377
## convergence code: 0
## boundary (singular) fit: see ?isSingular
```

Plot Data

```
plot.data <- analysis.female %>%
    group_by(ResponseId,sra) %>%
    summarise(
                mean.response = mean(response,na.rm = TRUE)
                ) %>%
```

```r
    filter(!is.na(mean.response))
plot.data2 <- group_by(plot.data,sra) %>%
    summarise(se = sd(mean.response,na.rm = TRUE)/sqrt(n()),
                mean.response = mean(mean.response,na.rm = TRUE))

ggplot(plot.data2,aes(x = sra,y = mean.response)) +
    stat_summary(fun.y=mean,geom="point") +
    geom_linerange(aes(ymin = mean.response - se,ymax = mean.response + se)) +
    geom_smooth(data = plot.data,method = "lm") +
    theme_classic() +
    xlab("Self-Rated Attractiveness") +
    ylab("Mean Masculinity Preference (with SE)")
```

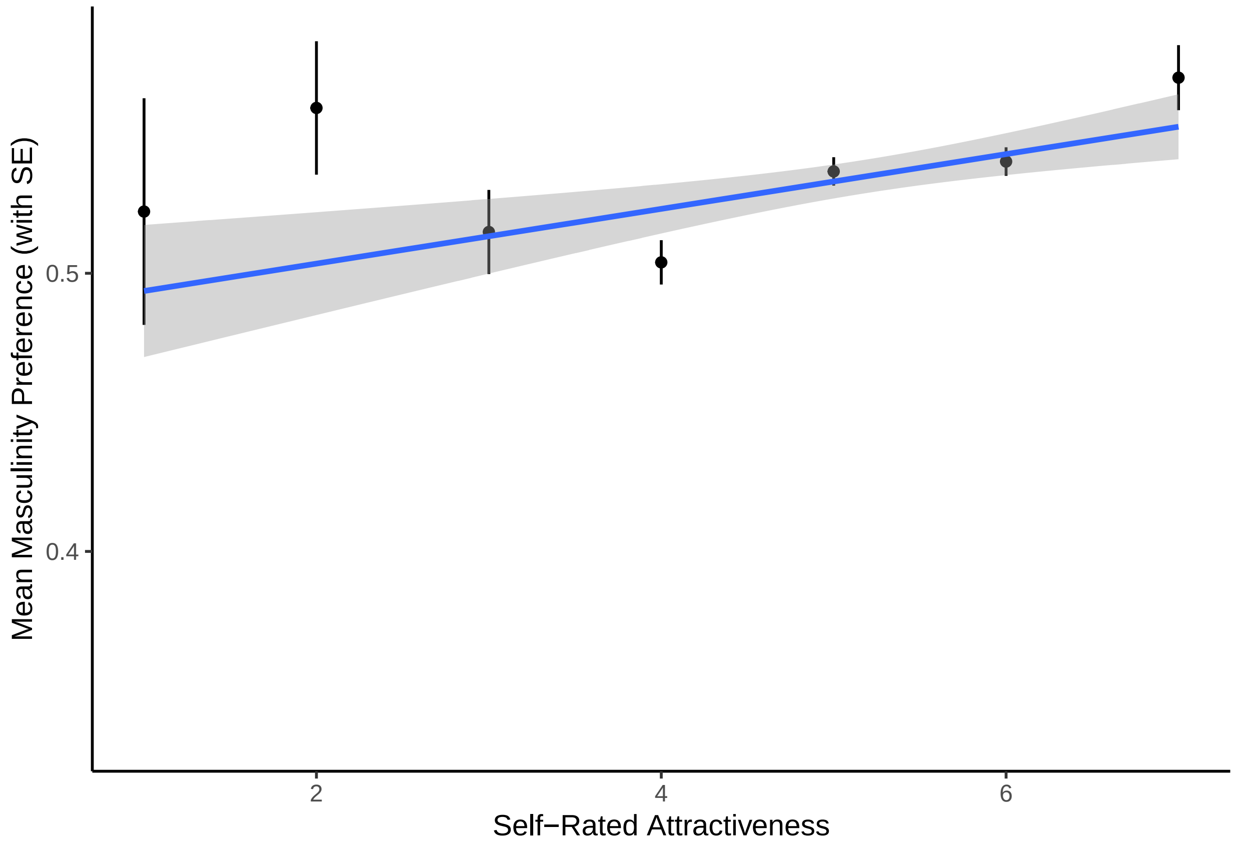


```r
ggsave(filename = "fig1.png")
```

Male Participants rating Female Faces

Linear Mixed Effects Model

```r
model.m <- glmer(response ~ z.age + z.sra + z.srh +
                    (1 | ResponseId) +
                    (1 + z.age + z.sra + z.srh || faceId) +
                    (1 + z.age + z.sra + z.srh || country) +
                    (1 + z.age + z.sra + z.srh || region),data = analysis.male,family = "binomial")
```

```r
save(model.m,file = "model.m.Rdata")
```

```r
load("model.m.Rdata")
summary(model.m)
```

```
## Generalized linear mixed model fit by maximum likelihood (Laplace
##   Approximation) [glmerMod]
##  Family: binomial  ( logit )
## Formula:
## response ~ z.age + z.sra + z.srh + (1 | ResponseId) + (1 + z.age +
##     z.sra + z.srh || faceId) + (1 + z.age + z.sra + z.srh ||
##     country) + (1 + z.age + z.sra + z.srh || region)
##    Data: analysis.male
##
##      AIC      BIC   logLik deviance df.resid
##  50507.6  50657.4 -25236.8  50473.6    49697
##
## Scaled residuals:
##     Min      1Q  Median      3Q     Max
## -6.1200 -0.5407  0.3858  0.5547  4.6970
##
## Random effects:
##  Groups     Name        Variance  Std.Dev.
##  ResponseId (Intercept) 4.395e-01 6.630e-01
##  country    z.srh       3.321e-09 5.763e-05
##  country.1  z.sra       1.907e-09 4.366e-05
##  country.2  z.age       1.016e-09 3.187e-05
##  country.3  (Intercept) 2.818e-02 1.679e-01
##  faceId     z.srh       0.000e+00 0.000e+00
##  faceId.1   z.sra       2.791e-03 5.283e-02
##  faceId.2   z.age       1.057e-02 1.028e-01
##  faceId.3   (Intercept) 1.035e+00 1.017e+00
##  region     z.srh       8.736e-05 9.347e-03
##  region.1   z.sra       1.217e-10 1.103e-05
##  region.2   z.age       5.919e-09 7.694e-05
##  region.3   (Intercept) 1.170e-01 3.421e-01
## Number of obs: 49714, groups:
## ResponseId, 2606; country, 41; faceId, 20; region, 7
##
## Fixed effects:
##             Estimate Std. Error z value Pr(>|z|)
## (Intercept)  0.95522    0.26942   3.545 0.000392 ***
## z.age       -0.11785    0.03057  -3.855 0.000116 ***
## z.sra        0.04965    0.02366   2.098 0.035898 *
## z.srh       -0.01281    0.05018  -0.255 0.798569
## ---
## Signif. codes:  0 '***' 0.001 '**' 0.01 '*' 0.05 '.' 0.1 ' ' 1
##
## Correlation of Fixed Effects:
##       (Intr) z.age  z.sra
## z.age  0.005
## z.sra -0.009 -0.006
## z.srh -0.021 -0.057 -0.188
## convergence code: 0
```

```
## boundary (singular) fit: see ?isSingular
```

Plot Data

```
plot.data <- analysis.male %>%
    group_by(ResponseId,sra) %>%
    summarise(
                mean.response = mean(response,na.rm = TRUE)
                ) %>%
    filter(!is.na(mean.response))
plot.data2 <- group_by(plot.data,sra) %>%
    summarise(se = sd(mean.response,na.rm = TRUE)/sqrt(n()),
                mean.response = mean(mean.response,na.rm = TRUE))

ggplot(plot.data2,aes(x = sra,y = mean.response)) +
    stat_summary(fun.y=mean,geom="point") +
    geom_linerange(aes(ymin = mean.response - se,ymax = mean.response + se)) +
    geom_smooth(data = plot.data,method = "lm") +
    theme_classic() +
    xlab("Self-Rated Attractiveness") +
    ylab("Mean Femininity Preference (with SE)")
```

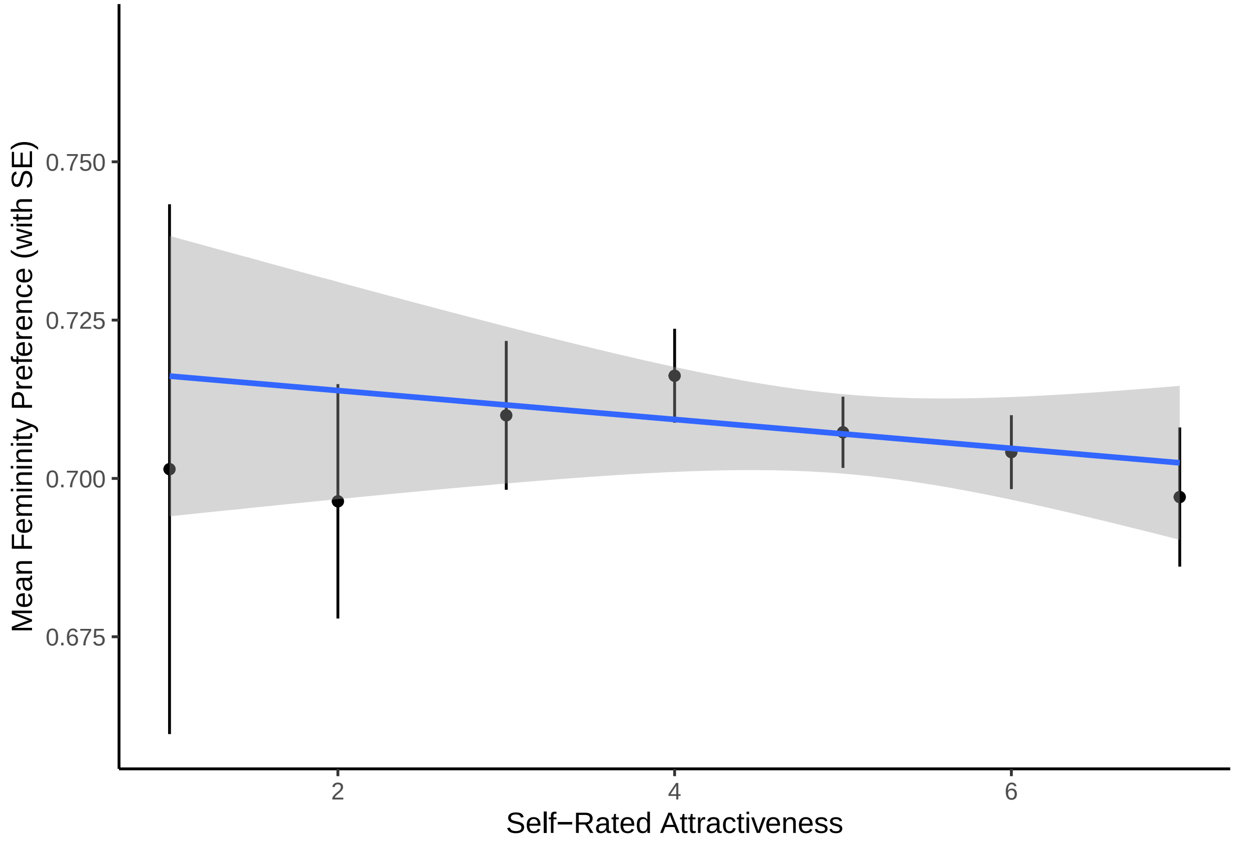

```r
ggsave("fig4.png")
```

Analysis with Ecological Factors

Female Participants rating Male Faces

Linear Mixed Effects Model

```r
model.f2 <- glmer(response ~ z.age*health.fa + z.sra*health.fa + z.srh*health.fa +
                    z.age*inequality.fa + z.sra*inequality.fa + z.srh*inequality.fa +
                    (1 + health.fa + inequality.fa || ResponseId) +
                    (1 + z.age:inequality.fa + z.sra:inequality.fa +
                       z.srh:inequality.fa + z.age:health.fa + z.sra:health.fa +
                       z.srh:health.fa || faceId) +
                    (1 + z.age + z.sra + z.srh || country) +
                    (1 + z.age:inequality.fa + z.sra:inequality.fa +
                       z.srh:inequality.fa + z.age:health.fa + z.sra:health.fa +
                       z.srh:health.fa || region),
                  data = analysis.female,family = "binomial")

save(model.f2,file = "model.f2.Rdata")
```

```r
load("model.f2.Rdata")
summary(model.f2)
```

```
## Generalized linear mixed model fit by maximum likelihood (Laplace
##   Approximation) [glmerMod]
##  Family: binomial  ( logit )
## Formula:
## response ~ z.age * health.fa + z.sra * health.fa + z.srh * health.fa +
##     z.age * inequality.fa + z.sra * inequality.fa + z.srh * inequality.fa +
##     (1 + health.fa + inequality.fa || ResponseId) + (1 + z.age:inequality.fa +
##     z.sra:inequality.fa + z.srh:inequality.fa + z.age:health.fa +
##     z.sra:health.fa + z.srh:health.fa || faceId) + (1 + z.age +
##     z.sra + z.srh || country) + (1 + z.age:inequality.fa + z.sra:inequality.fa +
##     z.srh:inequality.fa + z.age:health.fa + z.sra:health.fa +
##     z.srh:health.fa || region)
##    Data: analysis.female
##
##      AIC      BIC   logLik deviance df.resid
## 131290.6 131610.7 -65612.3 131224.6   120463
##
## Scaled residuals:
##     Min      1Q  Median      3Q     Max
## -8.1272 -0.6437  0.2236  0.6267  7.4748
##
## Random effects:
##  Groups       Name                Variance  Std.Dev.
##  ResponseId   inequality.fa       2.255e-08 1.502e-04
##  ResponseId.1 health.fa           0.000e+00 0.000e+00
##  ResponseId.2 (Intercept)         1.594e+00 1.263e+00
```

```
##  country      z.srh                4.970e-10 2.229e-05
##  country.1    z.sra                7.853e-09 8.862e-05
##  country.2    z.age                1.200e-02 1.095e-01
##  country.3    (Intercept)          4.537e-02 2.130e-01
##  faceId       z.srh:health.fa      1.512e-04 1.230e-02
##  faceId.1     z.sra:health.fa      5.253e-03 7.247e-02
##  faceId.2     z.age:health.fa      3.641e-02 1.908e-01
##  faceId.3     inequality.fa:z.srh 3.575e-04 1.891e-02
##  faceId.4     inequality.fa:z.sra 2.392e-04 1.547e-02
##  faceId.5     z.age:inequality.fa 4.453e-03 6.673e-02
##  faceId.6     (Intercept)          1.224e+00 1.106e+00
##  region       z.srh:health.fa      1.403e-10 1.185e-05
##  region.1     z.sra:health.fa      0.000e+00 0.000e+00
##  region.2     z.age:health.fa      0.000e+00 0.000e+00
##  region.3     inequality.fa:z.srh 0.000e+00 0.000e+00
##  region.4     inequality.fa:z.sra 0.000e+00 0.000e+00
##  region.5     z.age:inequality.fa 1.522e-10 1.234e-05
##  region.6     (Intercept)          1.612e-01 4.016e-01
## Number of obs: 120496, groups:
## ResponseId, 6325; country, 41; faceId, 21; region, 7
##
## Fixed effects:
##                       Estimate Std. Error z value Pr(>|z|)
## (Intercept)          0.0761328  0.3140568   0.242 0.808457
## z.age                0.1576698  0.0391176   4.031 5.56e-05 ***
## health.fa            0.2266693  0.0969417   2.338 0.019377 *
## z.sra                0.0758840  0.0208601   3.638 0.000275 ***
## z.srh                0.0003913  0.0204558   0.019 0.984736
## inequality.fa        0.1941969  0.0947811   2.049 0.040472 *
## z.age:health.fa     -0.0657335  0.0740501  -0.888 0.374708
## health.fa:z.sra      0.0253283  0.0319028   0.794 0.427240
## health.fa:z.srh     -0.0114122  0.0288689  -0.395 0.692613
## z.age:inequality.fa -0.0013402  0.0398198  -0.034 0.973150
## z.sra:inequality.fa  0.0087815  0.0218948   0.401 0.688364
## z.srh:inequality.fa -0.0120628  0.0213756  -0.564 0.572532
## ---
## Signif. codes:  0 '***' 0.001 '**' 0.01 '*' 0.05 '.' 0.1 ' ' 1
##
## Correlation of Fixed Effects:
##             (Intr) z.age  hlth.f z.sra  z.srh  inqlt. z.g:h. helth.f:z.sr
## z.age        0.009
## health.fa    0.215 -0.101
## z.sra        0.000  0.002  0.033
## z.srh       -0.010 -0.039  0.004 -0.432
## inequalty.f -0.175 -0.058  0.052 -0.018  0.020
## z.ag:hlth.f -0.017 -0.076  0.083  0.006  0.004  0.038
## helth.f:z.sr  0.025  0.002 -0.005 -0.045  0.047 -0.021  0.003
## hlth.f:z.srh  0.027  0.009  0.003  0.054 -0.112 -0.013 -0.041 -0.382
## z.g:nqlty.f -0.009 -0.054  0.068  0.011  0.001  0.067  0.394  0.002
## z.sr:nqlty.   0.008  0.004 -0.009  0.113 -0.042 -0.035  0.003  0.404
## z.srh:nqlt.   0.011  0.008 -0.006 -0.039  0.061 -0.002 -0.017 -0.187
##             hlth.f:z.srh z.g:n. z.sr:n.
## z.age
## health.fa
```

```
## z.sra
## z.srh
## inequalty.f
## z.ag:hlth.f
## helth.f:z.sr
## hlth.f:z.srh
## z.g:nqlty.f  -0.023
## z.sr:nqlty.  -0.200        0.011
## z.srh:nqlt.   0.475       -0.030 -0.433
## convergence code: 0
## boundary (singular) fit: see ?isSingular
```

Plot Data

Health/Development Factor

```
plot.data3 <- analysis.female %>%
  group_by(country,health.fa,inequality.fa,ResponseId) %>%
  summarise(meanMasc = mean(response,na.rm = TRUE)) %>%
  ungroup() %>%
  group_by(country,health.fa,inequality.fa) %>%
  summarise(mean = mean(meanMasc,na.rm = TRUE),
            se = sd(meanMasc,na.rm = TRUE)/sqrt(n()))

ggplot(plot.data3,aes(x = health.fa,y = mean)) +
  geom_pointrange(aes(ymin = mean - se,ymax = mean + se),size = .2) +
  geom_smooth(method = "lm") +
  xlab("Health/Development Factor") +
  ylab("Mean Masculinity Preference (with SE)") +
  theme_classic()
```

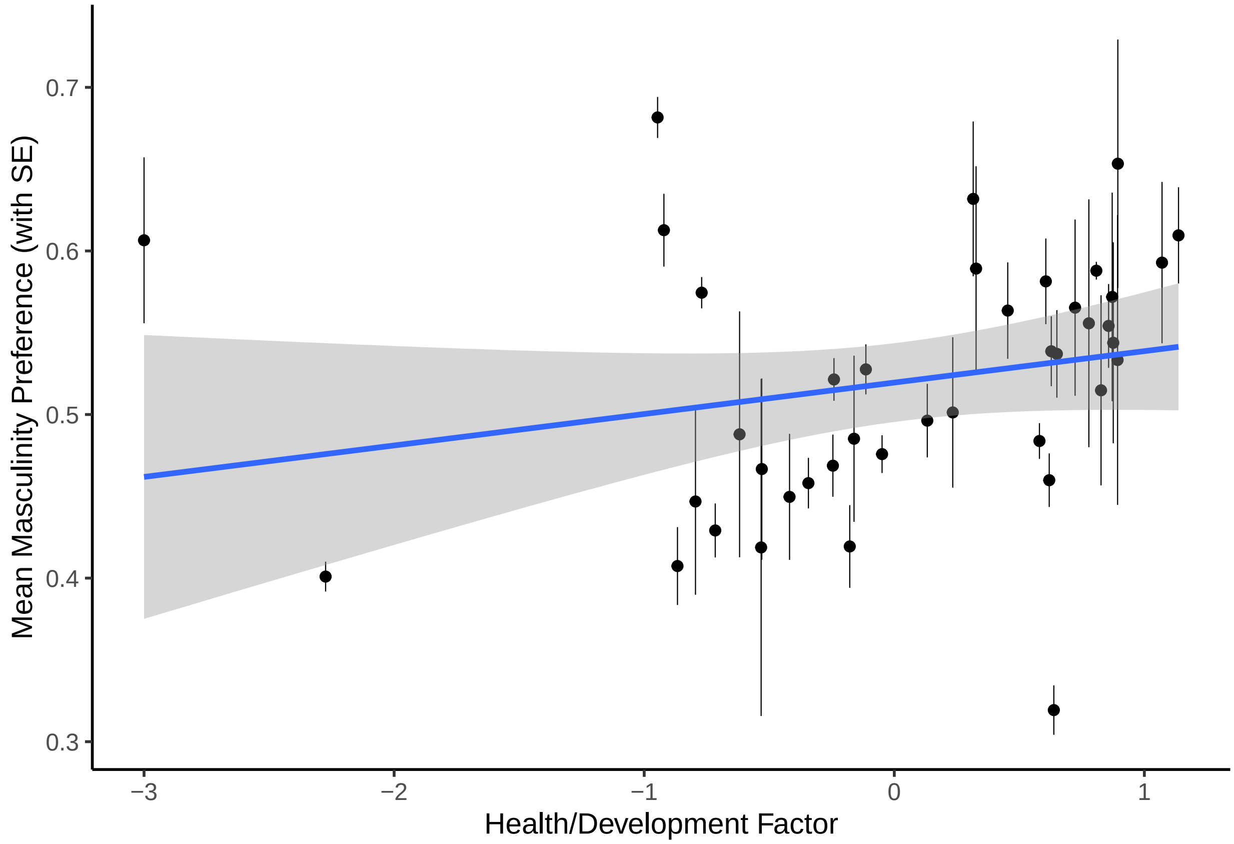


```
ggsave("fig2.png")
```

```
plot.data <- analysis.female %>%
    mutate(health.cat = ifelse(health.fa > 0,"High Health/Development","Low Health/Development"),
           inequality.cat = ifelse(inequality.fa > 0,"High Inequality","Low Inequality")) %>%
    group_by(ResponseId,sra,health.cat) %>%
    summarise(
                mean.response = mean(response,na.rm = TRUE)
                ) %>%
    filter(!is.na(mean.response))
plot.data2 <- group_by(plot.data,sra,health.cat) %>%
    summarise(se = sd(mean.response,na.rm = TRUE)/sqrt(n()),
                mean.response = mean(mean.response,na.rm = TRUE))

ggplot(plot.data2,aes(x = sra,y = mean.response,group = health.cat,colour = health.cat)) +
    geom_smooth(data = plot.data,method = "lm") +
    stat_summary(fun.y=mean,geom="point") +
    geom_linerange(aes(ymin = mean.response - se,ymax = mean.response + se)) +
    theme_classic() +
    xlab("Self-Rated Attractiveness") +
    ylab("Mean Masculinity Preference (with SE)") +
    labs(colour = "Health/Development")
```

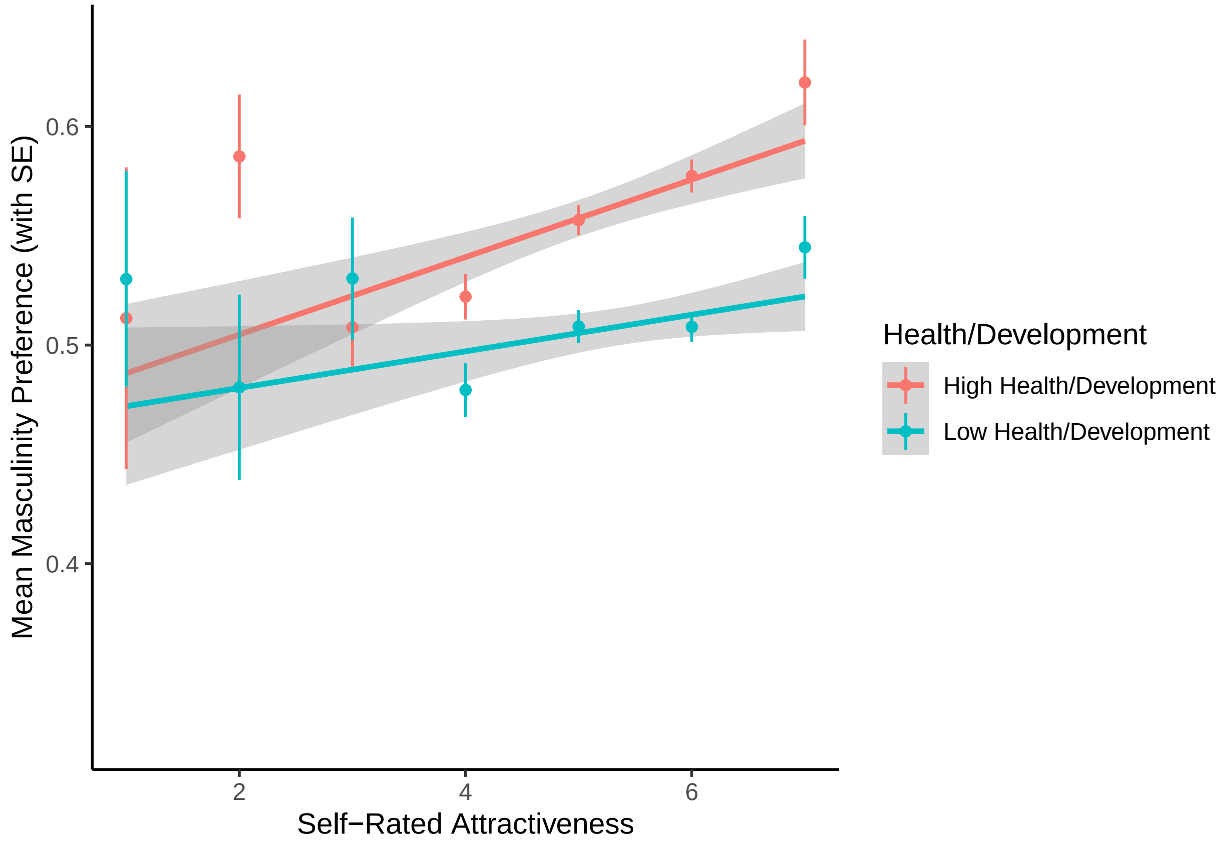

Inequality

```
ggplot(plot.data3,aes(x = inequality.fa,y = mean)) +
  geom_pointrange(aes(ymin = mean - se,ymax = mean + se),size = .2) +
  geom_smooth(method = "lm") +
  xlab("Inequality Factor") +
  ylab("Mean Masculinity Preference (with SE)") +
  theme_classic()
```

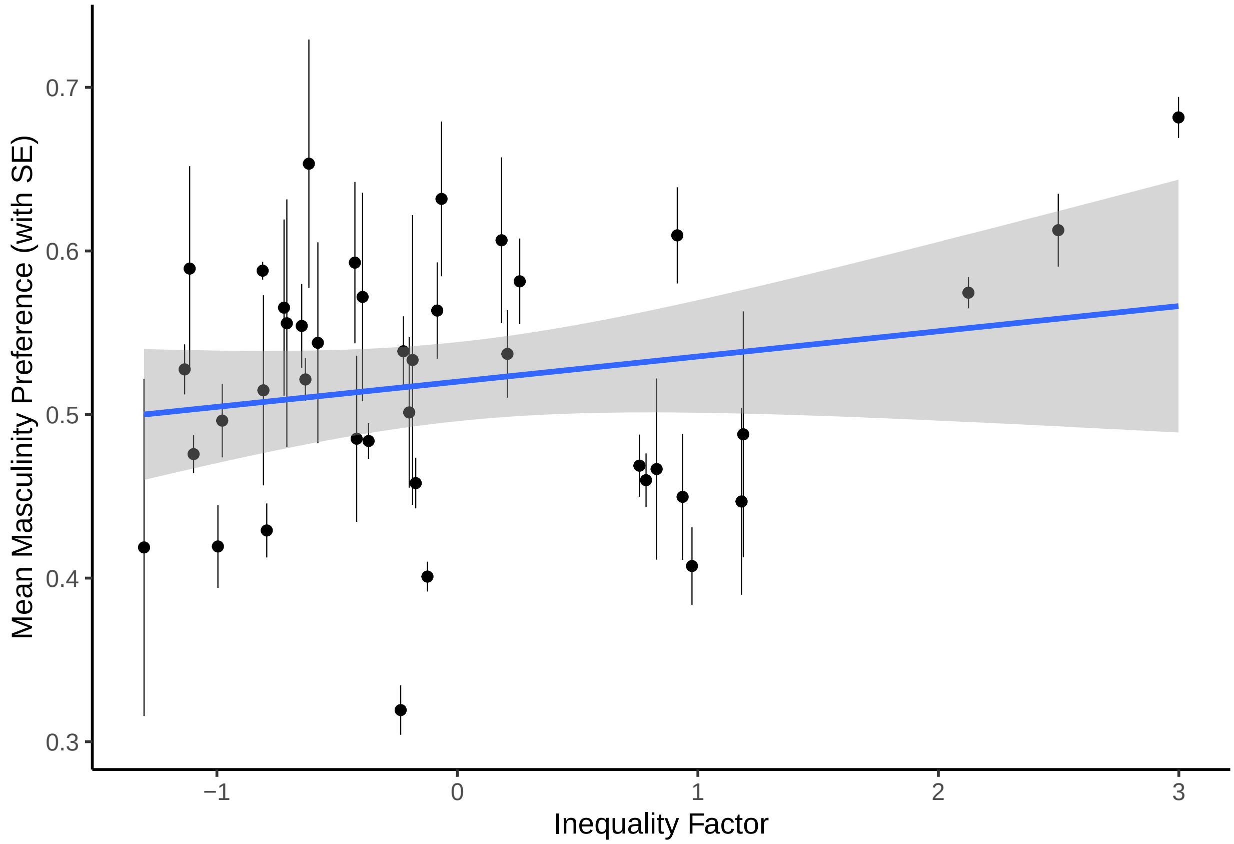


```
ggsave("fig3.png")
```

```
plot.data <- analysis.female %>%
    mutate(health.cat = ifelse(health.fa > 0,"High Health","Low Health"),
           inequality.cat = ifelse(inequality.fa > 0,"High Inequality","Low Inequality")) %>%
    group_by(ResponseId,sra,inequality.cat) %>%
    summarise(
                mean.response = mean(response,na.rm = TRUE)
                ) %>%
    filter(!is.na(mean.response))
plot.data2 <- group_by(plot.data,sra,inequality.cat) %>%
    summarise(se = sd(mean.response,na.rm = TRUE)/sqrt(n()),
                mean.response = mean(mean.response,na.rm = TRUE))

ggplot(plot.data2,aes(x = sra,y = mean.response,group = inequality.cat,colour = inequality.cat)) +
    geom_smooth(data = plot.data,method = "lm") +
    stat_summary(fun.y=mean,geom="point") +
    geom_linerange(aes(ymin = mean.response - se,ymax = mean.response + se)) +
    theme_classic() +
    xlab("Self-Rated Attractiveness") +
    ylab("Mean Masculinity Preference (with SE)") +
    labs(colour = "Inequality")
```

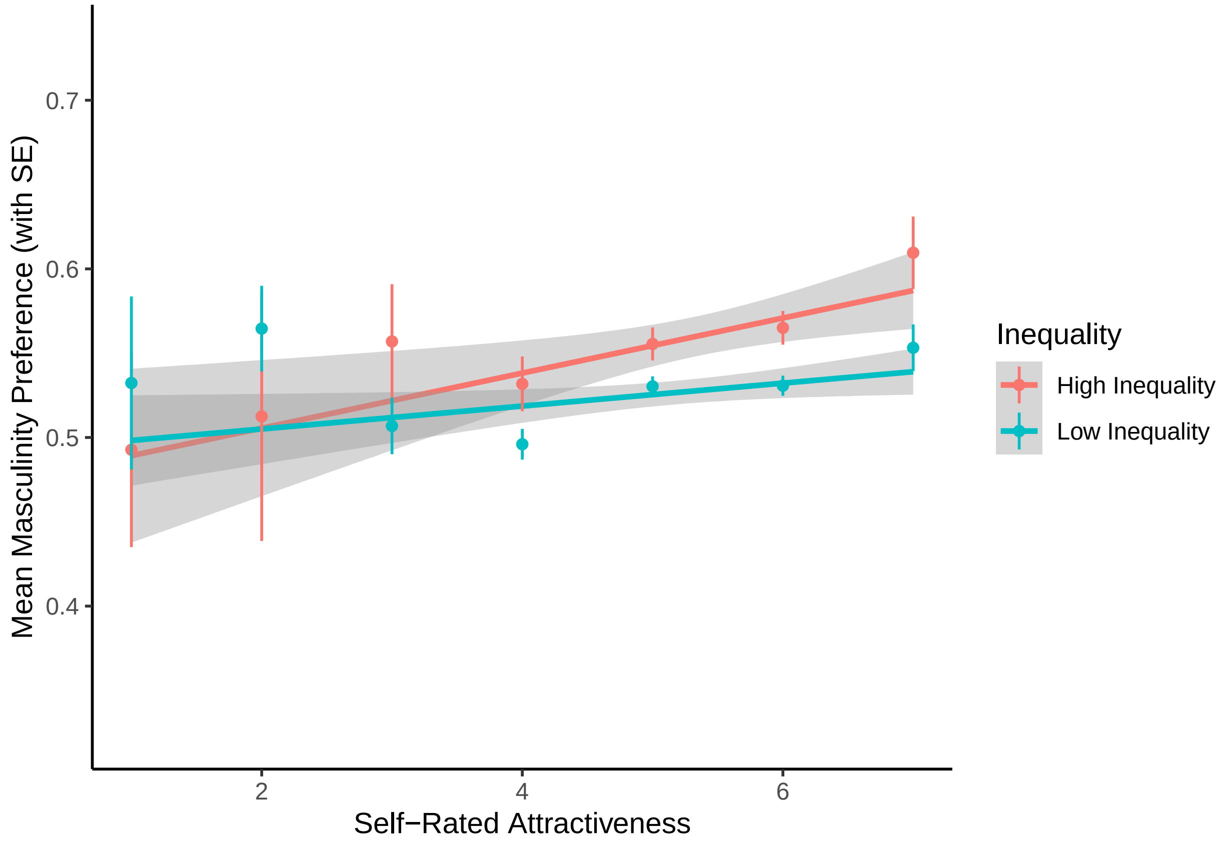

Male Analysis

Linear Mixed Effects Model

```
model.m2 <- glmer(response ~ z.age*health.fa + z.sra*health.fa + z.srh*health.fa +
                    z.age*inequality.fa + z.sra*inequality.fa + z.srh*inequality.fa +
                    (1 + health.fa + inequality.fa || ResponseId) +
                    (1 + z.age:inequality.fa + z.sra:inequality.fa +
                       z.srh:inequality.fa + z.age:health.fa + z.sra:health.fa +
                       z.srh:health.fa || faceId) +
                    (1 + z.age + z.sra + z.srh || country) +
                    (1 + z.age:inequality.fa + z.sra:inequality.fa +
                       z.srh:inequality.fa + z.age:health.fa + z.sra:health.fa +
                       z.srh:health.fa || region),
                  data = analysis.male,family = "binomial")

save(model.m2,file = "model.m2.2.Rdata")
```

```
load("model.m2.Rdata")
summary(model.m2)
```

```
## Generalized linear mixed model fit by maximum likelihood (Laplace
##   Approximation) [glmerMod]
##  Family: binomial  ( logit )
## Formula:
## response ~ z.age * health.fa + z.sra * health.fa + z.srh * health.fa +
```

```
##     z.age * inequality.fa + z.sra * inequality.fa + z.srh * inequality.fa +
##     (1 + health.fa + inequality.fa || ResponseId) + (1 + z.age:inequality.fa +
##     z.sra:inequality.fa + z.srh:inequality.fa + z.age:health.fa +
##     z.sra:health.fa + z.srh:health.fa || faceId) + (1 + z.age +
##     z.sra + z.srh || country) + (1 + z.age:inequality.fa + z.sra:inequality.fa +
##     z.srh:inequality.fa + z.age:health.fa + z.sra:health.fa +
##     z.srh:health.fa || region)
##    Data: analysis.male
##
##      AIC      BIC   logLik deviance df.resid
##  50430.8  50721.5 -25182.4  50364.8    49521
##
## Scaled residuals:
##     Min      1Q  Median      3Q     Max
## -5.1718 -0.5558 -0.3868  0.5442  6.4301
##
## Random effects:
##  Groups       Name                 Variance  Std.Dev.
##  ResponseId   inequality.fa        2.312e-02 1.521e-01
##  ResponseId.1 health.fa            3.728e-08 1.931e-04
##  ResponseId.2 (Intercept)          4.144e-01 6.438e-01
##  country      z.srh                2.167e-09 4.655e-05
##  country.1    z.sra                1.010e-09 3.179e-05
##  country.2    z.age                2.042e-03 4.519e-02
##  country.3    (Intercept)          1.541e-02 1.241e-01
##  faceId       z.srh:health.fa      2.537e-03 5.037e-02
##  faceId.1     z.sra:health.fa      3.893e-04 1.973e-02
##  faceId.2     z.age:health.fa      3.289e-03 5.735e-02
##  faceId.3     inequality.fa:z.srh 1.834e-08 1.354e-04
##  faceId.4     inequality.fa:z.sra 4.036e-09 6.353e-05
##  faceId.5     z.age:inequality.fa 2.411e-05 4.910e-03
##  faceId.6     (Intercept)          1.023e+00 1.011e+00
##  region       z.srh:health.fa      0.000e+00 0.000e+00
##  region.1     z.sra:health.fa      1.054e-08 1.027e-04
##  region.2     z.age:health.fa      1.243e-08 1.115e-04
##  region.3     inequality.fa:z.srh 7.168e-08 2.677e-04
##  region.4     inequality.fa:z.sra 2.558e-09 5.058e-05
##  region.5     z.age:inequality.fa 4.263e-10 2.065e-05
##  region.6     (Intercept)          3.768e-02 1.941e-01
## Number of obs: 49554, groups:
## ResponseId, 2598; country, 39; faceId, 20; region, 7
##
## Fixed effects:
##                      Estimate Std. Error z value Pr(>|z|)
## (Intercept)         -1.079467   0.248222  -4.349 1.37e-05 ***
## z.age                0.107760   0.022893   4.707 2.51e-06 ***
## health.fa           -0.145185   0.052961  -2.741  0.00612 **
## z.sra               -0.059842   0.020174  -2.966  0.00301 **
## z.srh                0.001629   0.020249   0.080  0.93588
## inequality.fa       -0.026891   0.068589  -0.392  0.69502
## z.age:health.fa      0.012851   0.027499   0.467  0.64027
## health.fa:z.sra     -0.053060   0.021822  -2.431  0.01504 *
## health.fa:z.srh      0.027139   0.024184   1.122  0.26178
## z.age:inequality.fa  0.021079   0.023282   0.905  0.36527
```

```
## z.sra:inequality.fa -0.029571   0.023346  -1.267  0.20530
## z.srh:inequality.fa -0.023073   0.023293  -0.991  0.32189
## ---
## Signif. codes:  0 '***' 0.001 '**' 0.01 '*' 0.05 '.' 0.1 ' ' 1
##
## Correlation of Fixed Effects:
##              (Intr) z.age  hlth.f z.sra  z.srh  inqlt. z.g:h. helth.f:z.sr
## z.age        -0.002
## health.fa     0.149 -0.001
## z.sra         0.001 -0.022 -0.001
## z.srh        -0.001 -0.037 -0.005 -0.449
## inequalty.f  -0.104  0.004  0.138 -0.002  0.000
## z.ag:hlth.f  -0.003  0.219 -0.009 -0.007 -0.015  0.001
## helth.f:z.sr  0.002  0.003  0.005  0.088 -0.027  0.001  0.027
## hlth.f:z.srh -0.001 -0.005 -0.008 -0.024  0.089 -0.003  0.037 -0.447
## z.g:nqlty.f   0.001  0.043 -0.001  0.000 -0.041 -0.009  0.183 -0.001
## z.sr:nqlty.   0.000 -0.006 -0.001  0.251 -0.118 -0.006  0.003  0.360
## z.srh:nqlt.  -0.001 -0.034 -0.003 -0.118  0.249  0.006  0.009 -0.179
##              hlth.f:z.srh z.g:n. z.sr:n.
## z.age
## health.fa
## z.sra
## z.srh
## inequalty.f
## z.ag:hlth.f
## helth.f:z.sr
## hlth.f:z.srh
## z.g:nqlty.f  -0.001
## z.sr:nqlty.  -0.162        0.012
## z.srh:nqlt.   0.327       -0.077 -0.474
## convergence code: 0
## boundary (singular) fit: see ?isSingular
```

Plot Data

Health/Development Factor

```
plot.data3 <- analysis.male %>%
  group_by(country,health.fa,inequality.fa,ResponseId) %>%
  summarise(meanMasc = mean(response,na.rm = TRUE)) %>%
  ungroup() %>%
  group_by(country,health.fa,inequality.fa) %>%
  summarise(mean = mean(meanMasc,na.rm = TRUE),
            se = sd(meanMasc,na.rm = TRUE)/sqrt(n()))

ggplot(plot.data3,aes(x = health.fa,y = mean)) +
  geom_pointrange(aes(ymin = mean - se,ymax = mean + se),size = .2) +
  geom_smooth(method = "lm") +
  xlab("Health/Development Factor") +
  ylab("Mean Femininity Preference (with SE)") +
  theme_classic()
```

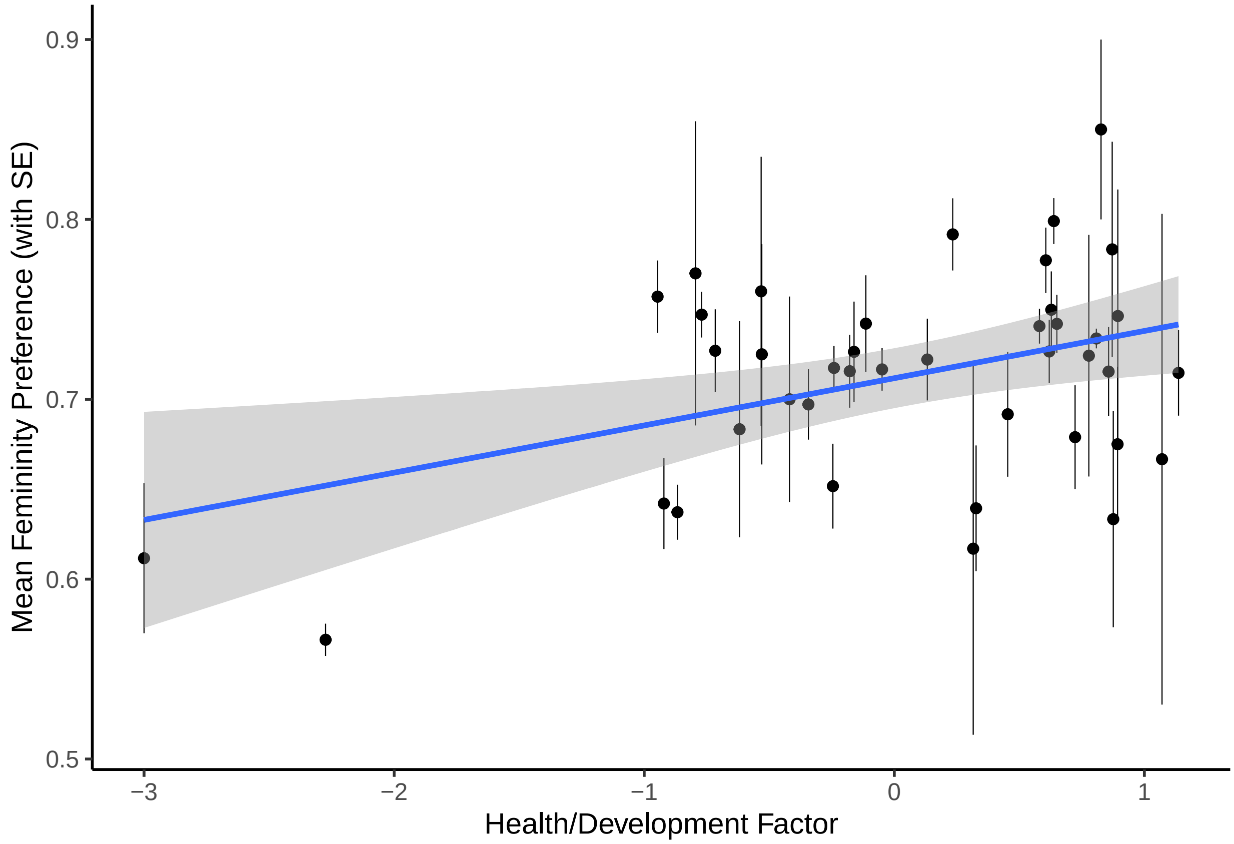


```
ggsave("Fig5.png")
```

```
plot.data <- analysis.male %>%
    mutate(health.cat = ifelse(health.fa > 0,"High Health/Development","Low Health/Development"),
            inequality.cat = ifelse(inequality.fa > 0,"High Inequality","Low Inequality")) %>%
    group_by(ResponseId,sra,health.cat) %>%
    summarise(
                mean.response = mean(response,na.rm = TRUE)
                ) %>%
    filter(!is.na(mean.response))
plot.data2 <- group_by(plot.data,sra,health.cat) %>%
    summarise(se = sd(mean.response,na.rm = TRUE)/sqrt(n()),
                mean.response = mean(mean.response,na.rm = TRUE))

ggplot(plot.data2,aes(x = sra,y = mean.response,group = health.cat,colour = health.cat)) +
    geom_smooth(data = plot.data,method = "lm") +
    stat_summary(fun.y=mean,geom="point") +
    geom_linerange(aes(ymin = mean.response - se,ymax = mean.response + se)) +
    theme_classic() +
    xlab("Self-Rated Attractiveness") +
    ylab("Mean Femininity Preference (with SE)") +
    labs(colour = "Health/Development")
```

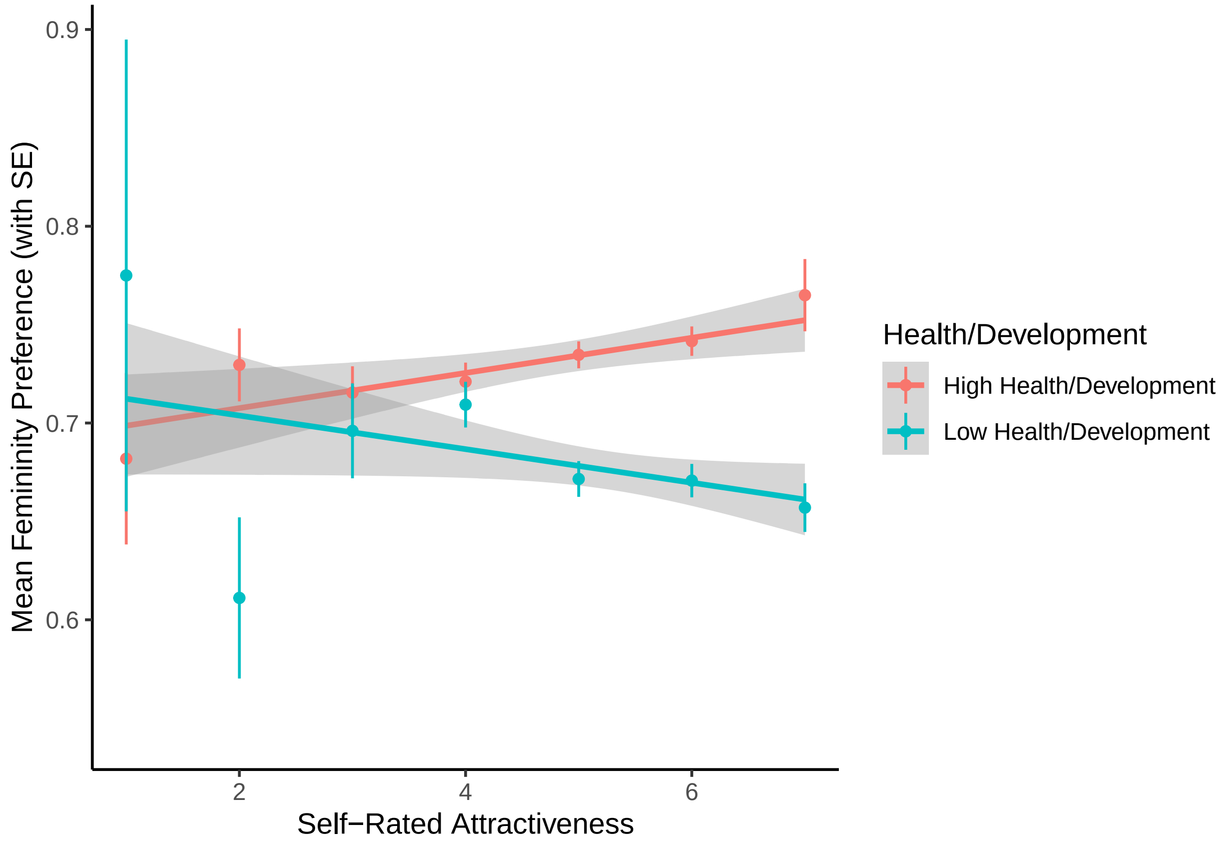


```
ggsave("fig6.png")
```

Inequality

```
ggplot(plot.data3,aes(x = inequality.fa,y = mean)) +
  geom_pointrange(aes(ymin = mean - se,ymax = mean + se),size = .2) +
  geom_smooth(method = "lm") +
  xlab("Inequality Factor") +
  ylab("Mean Femininity Preference (with SE)") +
  theme_classic()
```

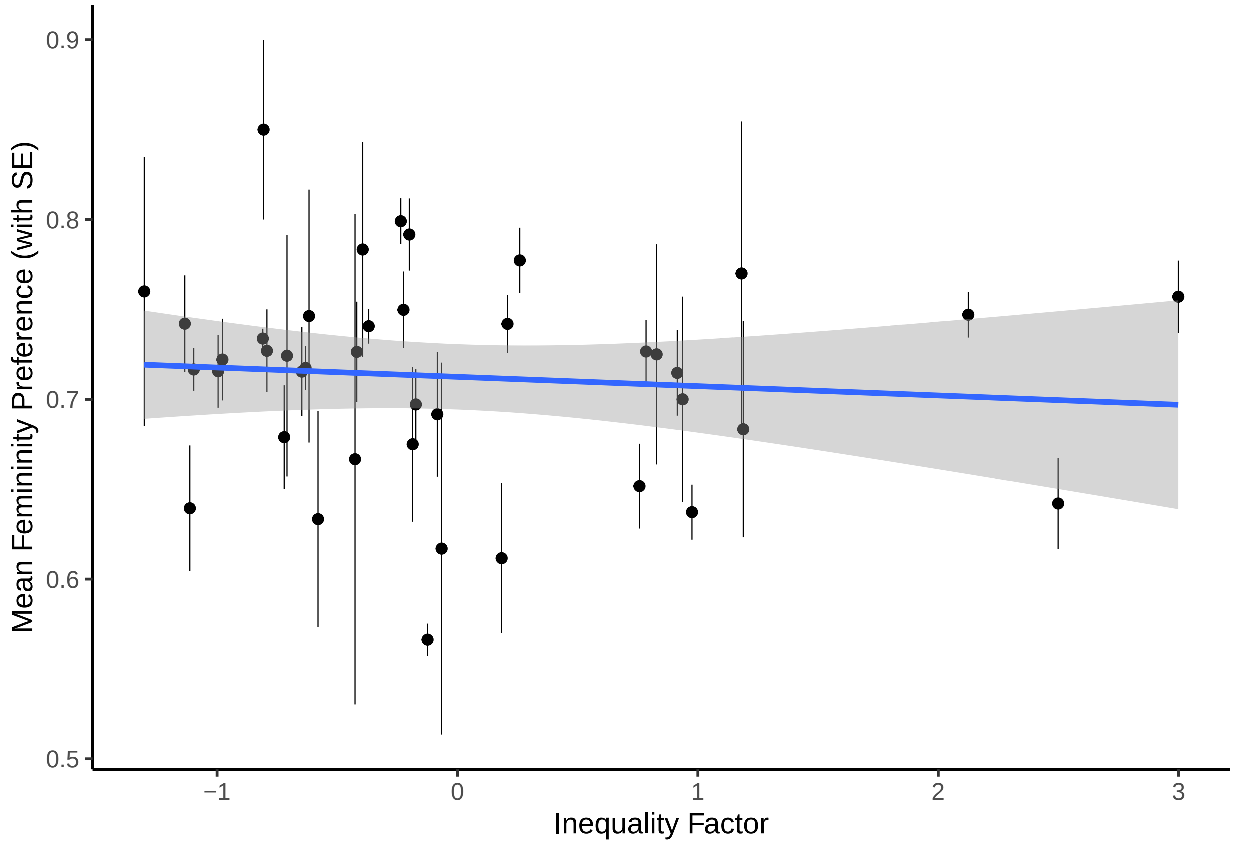


```
ggsave("fig3.png")
```

```
plot.data <- analysis.male %>%
    mutate(health.cat = ifelse(health.fa > 0,"High Health","Low Health"),
            inequality.cat = ifelse(inequality.fa > 0,"High Inequality","Low Inequality")) %>%
    group_by(ResponseId,sra,inequality.cat) %>%
    summarise(
                mean.response = mean(response,na.rm = TRUE)
                ) %>%
    filter(!is.na(mean.response))
plot.data2 <- group_by(plot.data,sra,inequality.cat) %>%
    summarise(se = sd(mean.response,na.rm = TRUE)/sqrt(n()),
                mean.response = mean(mean.response,na.rm = TRUE))

ggplot(plot.data2,aes(x = sra,y = mean.response,group = inequality.cat,colour = inequality.cat)) +
    geom_smooth(data = plot.data,method = "lm") +
    stat_summary(fun.y=mean,geom="point") +
    geom_linerange(aes(ymin = mean.response - se,ymax = mean.response + se)) +
    theme_classic() +
    xlab("Self-Rated Attractiveness") +
    ylab("Mean Femininity Preference (with SE)") +
    labs(colour = "Inequality")
```

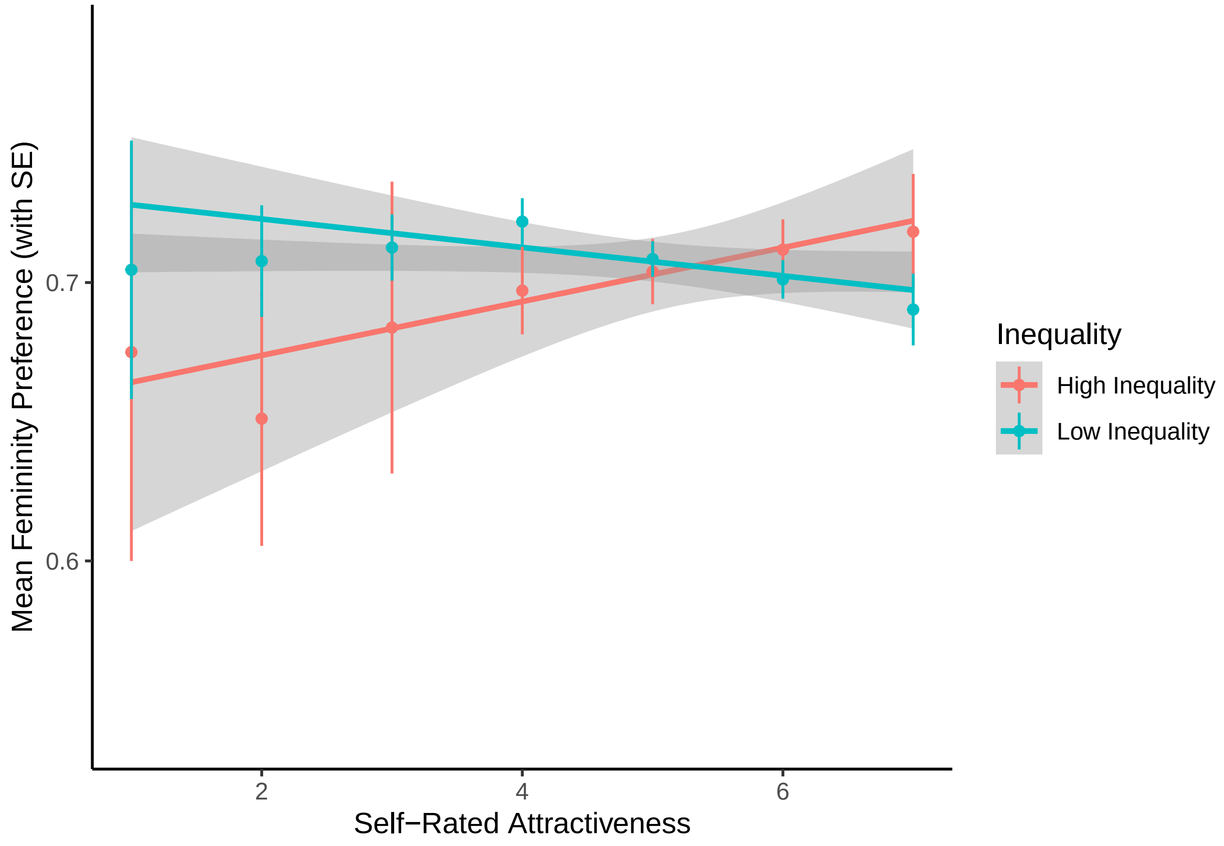
Mean Femininity Preference (with SE)
0.7
0.6
2
4
6
Self−Rated Attractiveness
Inequality
High Inequality
Low Inequality
